# Associations between sexual health and well-being: a systematic review

**DOI:** 10.2471/BLT.24.291565

**Published:** 2024-11-04

**Authors:** Priscila Vasconcelos, Mariana L Carrito, Ana Luísa Quinta-Gomes, Ana Luísa Patrão, Catarina AP Nóbrega, Pedro A Costa, Pedro J Nobre

**Affiliations:** a Centre for Psychology at University of Porto, Rua Alfredo Allen, s/n 4200-135, Porto, Portugal.

## Abstract

**Objective:**

To investigate the associations between sexual health dimensions, and overall health and well-being.

**Methods:**

In February 2024, we systematically searched Scopus, PsyArticles, PsycINFO®, PubMed®, Web of Science and LILACS for articles reporting on associations between sexual health, health and well-being indicators. We applied no language restrictions and followed the 2020 Preferred Reporting Items for Systematic Reviews and Meta-Analyses guidelines. To assess the risk of bias in the included studies, we used the Risk Of Bias In Non-randomized Studies - of Exposures tool.

**Findings:**

Of 23 930 unique titles identified, 63 studies met the inclusion criteria. We grouped the results into two categories: (i) sexual and physical health; and (ii) sexual and psychological health. The results consistently showed strong correlations between sexual health, overall health and well-being. Almost all studies found significant associations between positive sexual health indicators and lower depression and anxiety, higher quality of life, and greater life satisfaction among men and women, including older adults, pregnant women, and same-sex and mixed-sex couples.

**Conclusion:**

Findings indicate that emphasizing a positive perspective on sexual health and highlighting its benefits should be regarded as an important component of the effort to improve overall health and well-being for everyone.

## Résumé

**Objectif:**

Examiner les liens entre les composantes de santé sexuelle d'une part et, de l'autre, le bien-être et l'état de santé global.

**Méthodes:**

En février 2024, nous avons passé au crible Scopus, PsyArticles, PsycINFO®, PubMed®, Web of Science et LILACS à la recherche d'articles mentionnant des liens entre divers indicateurs relatifs à la santé sexuelle, la santé globale et le bien-être. Nous n'avons appliqué aucune restriction de langue et avons suivi les lignes directrices 2020 sur les éléments de rapport privilégiés dans le cadre des revues systématiques et méta-analyses. Enfin, pour déterminer le risque de biais dans les études sélectionnées, nous avons utilisé l'outil d'évaluation du risque de biais dans les études non randomisées d'exposition.

**Résultats:**

Sur les 23 930 titres uniques que nous avons identifiés, 63 études correspondaient aux critères d'inclusion. Nous avons réparti les résultats en deux catégories: (i) santé physique et sexuelle; et (ii) santé psychologique et sexuelle. Les résultats ont systématiquement montré de fortes corrélations entre santé sexuelle, santé globale et bien-être. Presque toutes les études ont observé des liens notables entre indicateurs de santé sexuelle positifs et moins de dépression et d'anxiété, une meilleure qualité de vie et davantage de satisfaction vis-à-vis de l'existence parmi les hommes et les femmes, y compris les personnes âgées et les femmes enceintes, tant chez les couples homosexuels qu'hétérosexuels.

**Conclusion:**

Ces résultats indiquent que mettre l'accent sur des perspectives positives en matière de santé sexuelle et souligner les avantages qui en découlent devrait être considéré comme un élément essentiel des efforts d'amélioration de la santé globale et du bien-être pour toutes et tous.

## Resumen

**Objetivo:**

Investigar las asociaciones entre las dimensiones de la salud sexual y la salud y el bienestar generales.

**Métodos:**

En febrero de 2024, se realizaron búsquedas sistemáticas en Scopus, PsyArticles, PsycINFO®, PubMed®, Web of Science y LILACS de artículos que informaran sobre asociaciones entre indicadores de salud sexual, salud y bienestar. No se aplicaron restricciones de idioma y se siguieron las directrices de 2020 sobre los elementos de informe preferidos para las revisiones sistemáticas y los metanálisis. Para evaluar el riesgo de sesgo en los estudios seleccionados, se utilizó la herramienta de evaluación del riesgo de sesgo en estudios no aleatorizados de exposición.

**Resultados:**

De los 23 930 títulos únicos identificados, 63 estudios cumplieron los criterios de inclusión. Se agruparon los resultados en dos categorías: (i) salud sexual y física; y (ii) salud sexual y psicológica. Los resultados indicaron de manera sistemática fuertes correlaciones entre la salud sexual, la salud y el bienestar generales. En la mayoría de los estudios, se hallaron asociaciones significativas entre los indicadores positivos de salud sexual y una menor depresión y ansiedad, una mayor calidad de vida y una mayor satisfacción vital entre hombres y mujeres, incluidos los adultos mayores, las mujeres embarazadas y las parejas del mismo sexo y mixtas.

**Conclusión:**

Los resultados indican que enfatizar una perspectiva positiva de la salud sexual y destacar sus beneficios debería considerarse un componente importante del esfuerzo por mejorar la salud y el bienestar general de todos.

## ملخص

**الغرض:**

استقصاء الارتباطات بين أبعاد الصحة الجنسية، والصحة العامة، والرفاهية.

**الطريقة:**

في فبراير/شباط 2024، قمنا بإجراء بحث منهجي في كل من Scopus، وPsyArticles، وPsycINFO®‎، وPubMed®‎، وWeb of Science، وLILACS، عن مقالات تعلّق على الارتباطات بين مؤشرات الصحة الجنسية، والصحة، والرفاهية. لم نقم بتطبيق أية قيود لغوية، واتبعنا المبادئ التوجيهية لعناصر التقارير المفضلة لعام 2020 للمراجعات المنهجية والتحليلات التلوية. لتقييم خطر التحيز في الدراسات المشمولة، استخدمنا أداة "خطر التحيز في الدراسات غير العشوائية - التعرض".

**النتائج:**

من بين 23930 عنوانًا فريدًا تم تحديدها، استوفت 63 دراسة معايير الاشتمال. قمنا بتجميع النتائج في فئتين: (أ) الصحة الجنسية والجسدية؛ و(ب) الصحة الجنسية والنفسية. أظهرت النتائج بشكل متسق ارتباطات قوية بين الصحة الجنسية، والصحة العامة، والرفاهية. وجدت جميع الدراسات تقريبًا ارتباطات ملموسة بين مؤشرات الصحة الجنسية الإيجابية، وانخفاض الاكتئاب والقلق، وارتفاع جودة الحياة، وزيادة الرضا عن الحياة بين الرجال والنساء، بما في ذلك كبار السن، والنساء الحوامل، والأزواج من نفس الجنس، والأزواج من جنسين مختلفين.

**الاستنتاج:**

تشير النتائج إلى أن التأكيد على منظور إيجابي للصحة الجنسية، وتسليط الضوء على فوائدها، يجب اعتباره مكونًا مهمًا للجهود المبذولة لتحسين الصحة العامة، والرفاهية للجميع.

## 摘要

**目的:**

旨在探讨性健康维度与整体健康和幸福感之间的关联性。

**方法:**

我们于 2024 年 2 月系统地检索了 Scopus、PsyArticles、PsycINFO®、PubMed®、Web of Science 和 LILACS，以获取报告性健康、健康和幸福感指标之间关联性的文章。我们并未就检索范围设置语言限制，并遵循了 2020 年《系统综述和荟萃分析优先报告条目》指南。为了评估纳入研究时存在的偏倚风险，我们使用了非随机暴露研究偏倚风险工具。

**结果:**

在检索到的 23,930 个独特标题中，有 63 项研究符合纳入标准。我们将结果分为两类：(i) 性健康和身体健康；以及 (ii) 性健康和心理健康。研究结果一致显示，性健康、整体健康和幸福感之间存在很强的关联性。几乎所有研究均表示，正向性健康指标与男性和女性（包括老年人、孕妇、同性和异性伴侣）的抑郁和焦虑程度降低、生活质量提高以及生活满意度提高之间存在明显相关性。

**结论:**

研究结果表明，强调积极看待性健康并重视其益处应被视为提高所有人整体健康水平和幸福感的重要工作要素。

## Резюме

**Цель:**

Изучить взаимосвязи между показателями сексуального здоровья и общим состоянием здоровья и благополучия.

**Методы:**

В феврале 2024 года был проведен систематический поиск статей в базах данных Scopus, PsyArticles, PsycINFO®, PubMed®, Web of Science и LILACS, в которых сообщалось о наличии взаимосвязей между сексуальным здоровьем, показателями здоровья и благополучия. Языковые ограничения не применялись, и в ходе исследования соблюдались рекомендации руководства «Предпочтительные компоненты для подготовки систематических обзоров и метаанализов», принятые в 2020 году. Для оценки риска необъективности включенных исследований использовался инструмент «Риск ошибки в нерандомизированных исследованиях» (Risk Of Bias In Non-randomized Studies - of Exposures).

**Результаты:**

Из 23 930 найденных уникальных названий 63 исследования соответствовали критериям включения. Полученные результаты разделены на две категории: (i) сексуальное и физическое здоровье; (ii) сексуальное и психологическое здоровье. Результаты свидетельствуют о сильной взаимосвязи между сексуальным здоровьем, общим состоянием здоровья и благополучием. Результаты большинства исследований свидетельствуют о значительной связи между положительными показателями сексуального здоровья и снижением уровня депрессии и тревожности, повышением качества жизни и удовлетворенности жизнью среди мужчин и женщин, включая пожилых людей, беременных женщин, а также однополые и разнополые пары.

**Вывод:**

Полученные данные свидетельствуют о том, что позитивное отношение к сексуальному здоровью и подчеркивание его преимуществ должны рассматриваться как важный компонент усилий по улучшению общего состояния здоровья и благополучия каждого человека.

## Introduction

The World Health Organization (WHO) defines sexual health as “a state of physical, emotional, mental and social well-being in relation to sexuality; it is not merely the absence of disease, dysfunction or infirmity.”^1^ The inclusion of sexual and reproductive health and rights in *Transforming our world: the 2030 agenda for sustainable development* has promoted sexual health as a priority in global public health, aiming to improve overall health throughout the lifespan.^2^ These advancements align with the recognition by WHO of sexual health as a fundamental human right,^3^ with sexual pleasure highlighted as a crucial component of sexual health and overall well-being throughout life.^4^^,^^5^

Sexuality can be affected by various health conditions, such as cardiovascular disease, mental health issues, menopause, age-related pathologies, neurological diseases, spinal cord injuries, combat injuries and cancer.^6^ Conversely, sexual health can positively affect health-related aspects, such as cardiovascular health.^7^^,^^8^ A positive prognosis of morbidity and mortality among diabetic patients has been associated with sexuality-related outcomes.^9^ The positive effect of sexual health is not only limited to physical health,^10^ but extends to subjective well-being^11^^,^^12^ and cognitive functioning.^13^^–^^15^ Given the evidence supporting sexual health's protective role in overall well-being, sexuality should be recognized as an inherent health factor, providing novel coping mechanisms, especially during challenging life stages such as adapting to chronic illness.^16^

Evidence of associations between sexual health and overall health and well-being could provide useful insights into the health benefits of being sexually healthy,^17^ framing sexual health as a (promotable) resource for protecting health and well-being. Considering the growing recognition of the importance of sexual health for physical and psychological health, and thus for personal fulfilment and well-being,^18^ we aimed to systematically identify studies analysing the associations between sexual health indicators and overall health and well-being.

## Methods

### Data search

We followed the Preferred Reporting Items for Systematic Reviews and Meta-Analyses guidelines^19^ and pre-registered the review in PROSPERO (CRD42024507701).^20^ In February 2024, we searched PsyArticles and PsycINFO® (both hosted by EBSCO), Scopus, PubMed®, Web of Science and LILACS for articles addressing the following questions: Does the sexual health of sexually active adults associate with their overall health and well-being? If so, what are the sexual health indicators linked to overall health and well-being? Are there differences in the subjective well-being of adults presenting different levels of sexual health? We used the WHO definition of sexual health.^1^ Other definitions considered are specified in Table 1.

**Table 1:** Definition of the outcomes included in the study on the association between sexual health and well-being

Outcome	Definition	Instruments
Health	Complete physical, mental, and social well-being, and not just the absence of disease^21^	Multiple instruments or constructs (e.g. Short form 36, Brief symptom inventory)
Quality of life	Quality of life refers to objective and subjective measures of physical, material, social and emotional well-being, as well as the level of personal growth and meaningful activity, weighed by individual sets of values.^22^ The WHO definition also comprehends an environmental dimension, which considers how safety, resources and living conditions affect quality of life^23^	The World Health Organization quality of life
Sexual distress	Negative emotional responses related to sexuality and sexual function^24^	Female sexual distress scale-revised
Sexual frequency	The frequency that the respondent engages in a specific sexual activity over a predetermined time period^25^	NA
Sexual function	The ease in progressing through the stages of sexual desire, arousal and orgasm, as well as feeling satisfied with the frequency and outcome of sexual activities^26^	International index of erectile function, Female sexual function index
Sexual satisfaction	Subjective evaluation of current sexual relationship^27^	New sexual satisfaction scale
Sexual well-being	Sexual well-being combines sexual health and sexual pleasure, reflecting high sexual satisfaction and reduced sexual distress, hence indicating an individual's perception of their sexual health^17^^,^^28^	Multiple instruments or constructs
Well-being	Well-being, experienced by either individuals or societies, is a positive state reflecting quality of life and the capacity to contribute to the world with meaning and purpose^29^	Multiple instruments or constructs (e.g. Satisfaction with life scale)

NA: not available; WHO: World Health Organization.

The data search strategy encompassed a holistic approach to sexual health, addressing not only sexual (dys)function but also positive aspects of sexual health.^30^ The search strategy had no language restrictions and included terms describing sexual health, health and well-being outcomes, combined with the connector “AND” to broad terms related to the topic of interest. The search string was (“sexual health” OR “sexual function” OR “sexual behavior” OR “sexual satisfaction” OR “sexual distress” OR “sexual well-being” OR “sexual pleasure”) AND (“health and well-being” OR “wellbeing” OR “wellness” OR “quality of life”).

### Eligibility criteria

The screening phase followed the predetermined review eligibility criteria according to the PICO framework: (P) studies using samples composed of men and/or women aged 18 years or older who have initiated their sexual lives; (I) studies designed to examine the associations between the psychosexual and behavioural components of sexual health, health and well-being indicators; (C) studies addressing differences in sexual health indicators, whenever possible (for example, studies comparing clinical samples to controls with no sexual complaints); and (O) studies using quantitative assessment of sexual health indicators (for example, sexual well-being, sexual function, sexual satisfaction, frequency of sexual activities and sexual distress) and overall health and well-being (for example, quality of life, satisfaction with life, anxiety and depression). Self-reported measures of health were also considered for inclusion. We excluded grey literature and studies including samples comprising only participants with physical comorbidities or addressing reproductive health or other dimensions of sexual and reproductive health (for example, sexually transmitted infections, harmful practices, sexual violence and access to health care). 

### Selection and data extraction

We retrieved identified studies stored in an EndNote (Clarivate, Philadelphia, United States of America) database. After removing duplicates, we downloaded the remaining records to the Rayyan platform (Rayyan, Cambridge, United States) to allow two authors to independently screen the data. The remaining authors resolved inclusion disagreements. First, the two authors determined if the title of each article met the predetermined eligibility criteria and analysed the abstract if the title alone was inconclusive. Subsequently, the authors examined if the abstracts and the full texts met the eligibility criteria. For eligible studies, we extracted data on the first author's name, publication year, country, sample characteristics, study design, sexual health measures, overall health measures, analytical approach and outcome results.

### Quality assessment

We used the Risk Of Bias In Non-randomized Studies - of Exposures tool^31^ to assess the risk of bias in the included studies, as the tool indicates whether the risk of bias is substantial enough to question the impact of the exposure on the outcome. *Robvis* was used to visualize the risk of bias results.^32^

## Results

**Fig. 1:**
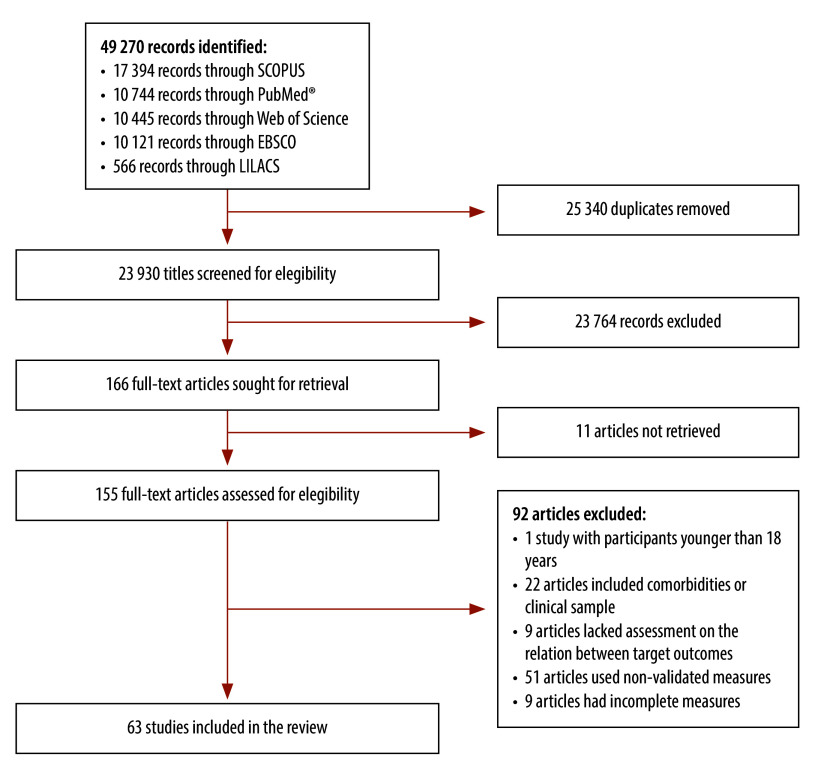
Flowchart of selection of studies on the associations between sexual health, overall health and well-being

The initial data search yielded 49 270 records. After eliminating duplicates, we screened 23 930 titles, followed by 377 abstracts. We retrieved 166 full-text reports and fully evaluated 155 for eligibility. Following full-text screening, we identified 63 studies eligible for inclusion (Fig. 1).^33^^–^^95^

**Fig. 2:**
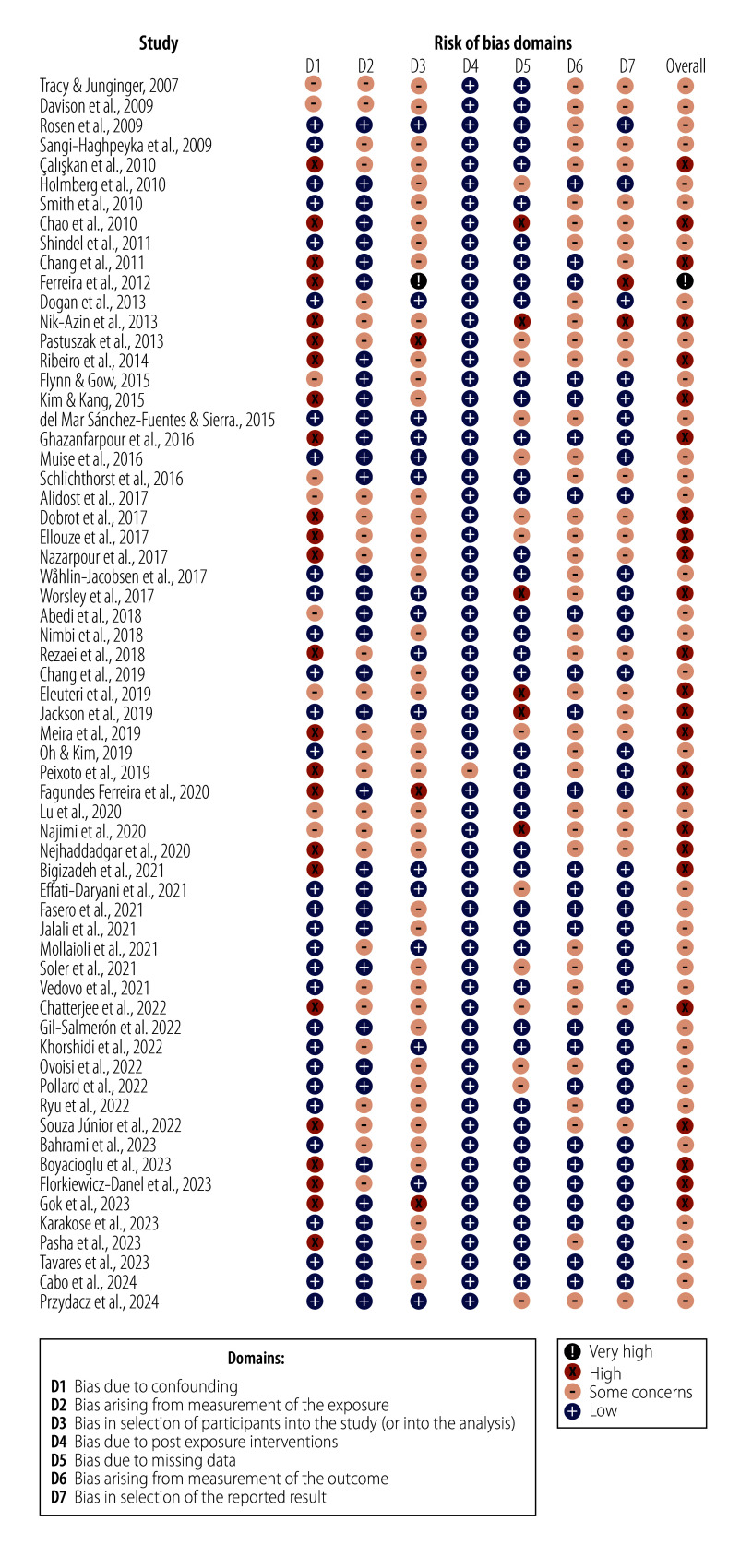
Quality assessment of studies included in the systematic review on associations between sexual health, overall health and well-being

**Fig. 3:**
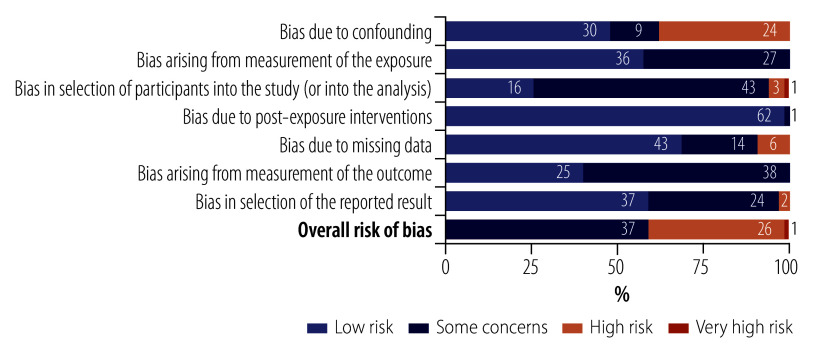
Distribution of biases across study components of studies included in the systematic review on associations between sexual health, overall health and well-being

Note: the numbers in the bars represent the number of studies.

Table 2 (available from: https://www.who.int/publications/journals/bulletin) presents data from the eligible studies. The sample size varied from 20 to 12 636 participants. Studies were conducted in five out of six WHO regions, with no studies from the African Region. Most studies were conducted in the Islamic Republic of Iran (14),^34^^,^^35^^,^^39^^,^^41^^,^^42^^,^^44^^,^^48^^,^^50^^,^^53^^,^^57^^,^^66^^,^^80^^,^^86^^,^^91^ United States of America (10)^37^^,^^40^^,^^62^^,^^67^^,^^68^^,^^73^^,^^77^^,^^85^^,^^92^^,^^95^ and Brazil (6).^36^^,^^38^^,^^43^^,^^51^^,^^58^^,^^72^ The age of the participants ranged from 18 to 75 years and older. Sixty-one studies applied cross-sectional research designs, while two studies conducted longitudinal research. Most studies focused on sexual functioning and distress, using scales such as the Female Sexual Function Index^96^ (27 studies);^34^^–^^36^^,^^39^^–^^47^^,^^50^^–^^53^^,^^66^^,^^68^^,^^69^^,^^71^^–^^74^^,^^82^^,^^89^^–^^91^ the original and short versions of the International Index of Erectile Function^97^^,^^98^ (9 studies);^59^^,^^60^^,^^62^^,^^75^^–^^78^^,^^82^^,^^90^ and the original and revised versions of the Female Sexual Distress Scale^99^^,^^100^ (5 studies).^37^^,^^69^^,^^86^^,^^89^^,^^90^ Studies employed multidimensional approaches to measuring health and well-being,^101^ such as applying different versions of the World Health Organization Quality of Life measurement tool^23^^,^^102^^,^^103^ (12 studies)^33^^,^^34^^,^^36^^,^^38^^,^^39^^,^^53^^–^^56^^,^^58^^,^^61^^,^^76^ and the 12-item and 36-item Short Form Health Surveys^104^^,^^105^ (9 studies).^43^^–^^46^^,^^51^^,^^52^^,^^63^^,^^65^^,^^75^The assessment of the risk of bias showed that most studies had some degree of bias. The most considerable concerns were confounding bias and selection bias, primarily due to not adjusting for confounding factors, using non-probabilistic sampling methods and lack of information on blinding practices. (Fig. 2 and Fig. 3).

**Table 2:** Summary of the characteristics of the included studies in the systematic review on sexual health and well-being

Study	Country	Sample characteristics	Study design	Sexual health measures	Health and well-being measures
Tracy & Junginger, 2007^73^	USA	350 women; mean age 35.5 years (SD: 11.4; range: 18–73)	Cross-sectional	Female Sexual Function Index-modified version	Brief Symptom Inventory
Davison et al., 2009^93^	Australia	421 women aged 18–65 years; mean age of premenopause group 39.6 years (SD: 6.7); postmenopause group: 55.5 (SD: 5.4)	Cross-sectional	Psychological General Well-being Index and the Beck Depression Index (at baseline); Daily diary of sexual frequency	Psychological General Well-being Index and Beck Depression Inventory
Rosen et al., 2009^37^	USA	31 581 women^e^ aged 18–75 years	Cross-sectional	Changes in Sexual Functioning Questionnaire Short-Form and Female Sexual Distress Scale-12	12-Item Short Form Survey
Sangi-Haghpeyka et al., 2009^40^	USA	138 women 137 men; mean age 29.3 years	Cross-sectional	Male Sexual Function Inventory and, Female Sexual Function Index	Quality of Life Inventory-36 and Stress Inventory
Çaliskan et al., 2010^33^	Türkiye	300 women aged 40–50 years	Cross-sectional	Golombok Rust Inventory of Sexual Satisfaction	WHO Quality of Life Brief Version
Holmberg et al., 2010^64^	Canada	322 women; mean age for women in mixed-sex relationship 26.0 years (SD: 6.65; range: 18–55); for same sex-relationship 33.6 (SD: 9.77; range 18–58)	Cross-sectional	Index of Sexual Satisfaction and Sexual Satisfaction Inventory	Cohen–Hoberman Inventory of Physical Symptoms, Centre for Epidemiological Studies Depression Scale,-20, State–Trait Anxiety Inventory and 10-item Perceived Stress Scale
Smith et al., 2010^67^	USA	844 men; mean age 25.7 years (SD: 4.1)	Cross-sectional	International Index of Erectile Function and Premature Ejaculation Diagnostic Tool	Center for Epidemiologic Studies Depression Scale
Chao et al., 2011^61^	China, Taiwan	83 women and 200 men aged ≥ 45 years	Cross-sectional	Sexual Desire Inventory and Sexual Satisfaction Scale	WHO Quality of Life Assessment
Shindel et al., 2011^68^	USA	1241 women; mean age 25.4 years (SD: 3.4)	Cross-sectional	Female Sexual Function Index	Center for Epidemiologic Studies Depression Scale
Chang et al., 2012^74^	China, Taiwan	555 women aged ≥ 18 years; mean age 32.95 years (SD: 0.16)	Cross-sectional	Female Sexual Function Index	Center for Epidemiologic Studies Depression Scale
Ferreira et al., 2012^38^	Brazil	51 women; mean age 26.9 years (SD: 5.3; range: 20–37)	Cross-sectional	Sexual Quotient-Female version	WHO Quality of Life Brief Version
Dogan et al., 2013^94^	Türkiye	204 women; mean age 31.98 years (range: 17–63)	Cross-sectional	Sexual Quality of Life Questionnaire-Female version	Satisfaction with Life Scale and Oxford Happiness Questionnaire-Short Form
Nik-Azin et al., 2013^35^	Islamic Republic of Iran	150 women; mean age 28.4 years (SD: 4.96)	Cross-sectional	Female Sexual Function Index	Depression Anxiety and Stress Scale −21 and WHO Quality of Life Brief Version
Pastuszak et al., 2013^77^	USA	186 men; mean age 52.6 years (SD: 12.7)	Cross-sectional	International Index of Erectile Function	Patient Health Questionnaire-9
Ribeiro et al., 2014^72^	Brazil	152 women, age not reported	Cross-sectional	Female Sexual Function Index	Beck Depression Inventory
Flynn & Gow, 2015^55^	United Kingdom	62 women and 71 men; mean age 74 years (SD: 7.1; range: 65–92)	Cross-sectional	Sexual Behaviour Frequency Scale and participants were asked to rate the same sexual behaviours in terms of importance	WHO Quality of Life Brief Version
Kim & Kang, 2015^56^	Republic of Korea	186 women and 181 men; mean age 52.77 years (SD: 4.5; range: 45–60)	Cross-sectional	Sexual Quality of Life Questionnaire	Centre for Epidemiological Studies Depression Scale and WHO Quality of Life Brief Version
del Mar Sánchez-Fuentes & Sierra, 2015^65^	Spain	1 009 women and 1 015 men^d^ aged 18–80 years	Cross-sectional	Global Measure of Sexual Satisfaction	Short Form 36, Symptom Assessment-45 Questionnaire
Ghazanfarpour et al., 2016^50^	Islamic Republic of Iran	202 women;^c^ mean age 52.69 years (SD: 37; range: 40–70)	Cross-sectional	Female Sexual Function Index	Menopause-Specific Quality of Life
Muise et al., 2016^84^	Canada	197 women and 138 men;^d^ mean age 31 years (SD: 9; range: 18–64)	Cross-sectional	Sexual frequency	Satisfaction with Life Scale
Schlichthorst et al., 2016^63^	Australia	12 636 men;^e^ mean age 35.0 years (SD: 10; range: 18–55)	Longitudinal	National Survey of Sexual Attitudes and Lifestyles sexual function questionnaire-3	12-Item Short Form Survey
Alidost et al., 2017^53^	Islamic Republic of Iran	300 women; mean age 27.38 years (SD: 5.49; range: 16–43)	Cross-sectional	Female Sexual Function Index	WHO Quality of Life Brief Version and Prenatal Anxiety Questionnaire
Debrot et al., 2017^92^ (study 1)	USA	138 men and 197 women; mean age 31 years (SD: 9.1; range:18–64)	Cross-sectional	Sexual frequency: participants indicated how frequently they engaged in sex with their partner. Affectionate touch frequency: participants indicated the general frequency of affectionate touch (e.g. cuddling, kissing, caressing) in their relationship.	Satisfaction with Life Scale
Ellouze et al., 2017^46^	Tunisia	100 women; mean age 29.4 years (SD: 5.6)	Cross-sectional	Female Sexual Function Index	Edinburgh Postnatal Depression Scale and 12-Item Short Form Survey
Nazarpour et al., 2017^39^	Islamic Republic of Iran	405 women; mean age 52.8 years (SD: 3.7)	Cross-sectional	Female Sexual Function Index	WHO Quality of Life Brief Version
Wåhlin-Jacobsen et al., 2017^89^	Denmark	428 women aged 19–58 years	Cross-sectional	Female Sexual Function Index and Female Sexual Distress Scale	Beck Depression Inventory-II
Worsley et al., 2017^69^	Australia	2020 women; mean age 52.6 years (SD: 6.8; range 40–65)	Cross-sectional	Female Sexual Function Index; Female Sexual Distress Scale-revised	Beck Depression Inventory-II
Abedi et al., 2018^66^	Islamic Republic of Iran	1200 women^a^; mean age 30.76 years (SD: 7.14; range:15–45)	Cross-sectional	Female Sexual Function Index	Health Promoting Lifestyle Profile 2
Nimbi et al., 2018^75^	Italy	298 men; mean age 32.66 years (SD:11.52; range 18–72)	Cross-sectional	International Index of Erectile Function, Premature Ejaculation Severity Index, Sexual Distress Scale-Male, Sexual Satisfaction Scale-Male and Sexual Modes Questionnaire	Short Form 36, Beck Depression Inventory, State–Trait Anxiety Inventory Form Y, Symptom Check List-90-Revised and Toronto Alexithymia Scale-20
Rezaei et al., 2018^44^	Islamic Republic of Iran	380 women aged ≥ 18 years;^b^ mean age 29.81 years (SD: 5.5)	Cross-sectional	Female Sexual Function Index	Short Form 36
Chang et al., 2019^45^	China, Taiwan	1026 women; mean age 48.51 years (SD: 0.17; range: 40–65)	Cross-sectional	Female Sexual Function Index	12-Item Short Form Survey
Eleuteri et al., 2019^59^	Italy	40 men; mean age 75.4 years (SD: 7.3)	Cross-sectional	International Index of Erectile Function-5	Beck Depression Inventory, Mini Mental State Examination and Quality of Life Index
Jackson et al., 2019^80^	United Kingdom	3217 women and 2614 men aged ≥ 50 years;^e^ mean age 68.4 years (SD: 9.95)	Cross-sectional	Frequency of sexual activities	Satisfaction with Life Scale, Center for Epidemiologic Studies Depression Scale 8 and Control, Autonomy, Self-Realization and Pleasure–19
Meira et al., 2019^36^	Brazil	20 women; aged 38–60 years	Cross-sectional	Female Sexual Function Index	WHO Quality of Life Brief Version
Oh & Kim, 2019^47^	Republic of Korea	138 women; mean age 32.62 years (SD: 4.27; range: 22–43)	Cross-sectional	Female Sexual Function Index-6 item Korean version	EuroQol-5 Dimension
Peixoto et al., 2019^51^	Brazil	36 women; mean age 55.39 years (SD: 4.68; range: 45–65)	Cross-sectional	Female Sexual Function Index	Short Form 36
Fagundes Ferreira et al., 2020^43^	Brazil	278 women; mean age 32 years (SD: 5.60; range: 18–40)	Cross-sectional	Female Sexual Function Index	Short Form 36
Lu et al., 2020^60^	China	1267 men; mean age 59.09 years (SD: 8.65; range: 50–70)	Cross-sectional	International Index of Erectile Function-5 and Premature Ejaculation Diagnostic Tool	General Anxiety Disorder-7, Patient Health Questionnaire-9, Satisfaction with Life Scale and Control, Autonomy, Self-Realization and Pleasure-19
Najimi et al., 2020^57^	Islamic Republic of Iran	362 men; mean age 69.9 years (SD: 8.1; range: 60–100)	Cross-sectional	Sexual Quality of Life Questionnaire-Male	General Health Questionnaire-28
NeJhaddadgar et al., 2020^41^	Islamic Republic of Iran	1245 women; mean age 75.1 years (SD: 7.2; range: 60–87)	Cross-sectional	Female Sexual Function Index	General Health Questionnaire
Bigizadeh et al., 2021^34^	Islamic Republic of Iran	318 women; mean age 20.78 years (SD: 4.23)	Cross-sectional	Female Sexual Function Index	WHO Quality of Life Brief Version
Effati-Daryani et al., 2021^71^	Islamic Republic of Iran	437 women;^b^ mean age 29.7 years (SD: 3.3; range: 19–44)	Cross-sectional	Female Sexual Function Index	Depression Anxiety and Stress Scale-21
Fasero et al., 2021^49^	Spain	521 women; mean age 51.3 years (SD: 4.9; range 45–65)	Cross-sectional	Brief Profile of Female Sexual Function	Cervantes-Short Form Scale
Jalali et al., 2021^48^	Islamic Republic of Iran	558 women; mean age 54.01 years (SD: 3.95; range: 40–60)	Cross-sectional	Sexual Self-Efficacy Scale	Menopause-Specific Quality of Life
Mollaioli et al., 2021^82^	Italy	4 177 women and 2 644 men; mean age 32.83 years (SD: 11.24)	Cross-sectional	International Index of Erectile Function-15 and Female Sexual Function Index	General Anxiety Disorder-7 and Patient Health Questionnaire-9
Soler et al., 2021^70^	Spain	700 women and 516 men; mean age 21.4 years (SD: 3.42; range: 18–35)	Cross-sectional	Massachusetts General Hospital Sexual Functioning Questionnaire	Symptom Assessment-45 Questionnaire
Vedovo et al., 2021^52^	Italy	122 women; mean age transgender women 38.5 years (SD: 9.2); cisgender women: 37.7 (SD: 11.5)	Cross-sectional	Female Sexual Function Index; operated Male to Female Sexual Function Index	Beck Depression Inventor Primary Care and Short Form 36
Chatterjee et al., 2022^54^	India	1 108 men and 268 women aged ≥ 19 years; mean age 34.42 years (SD: 9.34)	Cross-sectional	Arizona Sexual Experiences scale	Depression Anxiety and Stress Scale and WHO Quality of Life Brief Version
Gil-Salmerón et al., 2022^81^	Spain	220 women and 85 men aged 18–74 years	Cross-sectional	Sexual frequency	Beck Anxiety Inventory and Beck Depression Inventory
Khorshidi et al., 2022^86^	Islamic Republic of Iran	536 women;^b,c^ mean age 36.75 years (SD: 7.48; range: 18–59)	Cross-sectional	Female Sexual Quality of Life Questionnaire and Female Sexual Distress Scale-revised and sexual frequency per month	Psychological Distress Scale
Oveisi et al., 2022^88^	Canada	124 women ≥ 18 years, mean age 21 years (SD: 2)	cross-sectional	Sexual Quality of Life Questionnaire-Female	Mental Health Continuum Short Form
Pollard; 2022^95^	USA	1241; Aged 18–80 years; 47 nonbinary, fluid or other people, 775 women and 419 men	Cross-sectional	Quality of Sex Index-6	Patient Health Questionnaire-9
Ryu et al., 2022^76^	Republic of Korea	216 men; mean age 50.09 years (SD: 6.29; range: 41–64)	Cross-sectional	International Index of Erectile Function	Beck Depression Inventory-Korean Version, Sherer's General Self-Efficacy Scale and WHO Quality of Life Brief Version
de Souza Júnior et al., 2022^58^	Brazil	231 men aged ≥ 60 years	Cross-sectional	Male Sexual Quotient and *Escala de Vivências Afetivas e Sexuais do Idoso*	World Health Organization Quality of Life Assessment for Older Adults
Bahrami et al., 2023^91^	Islamic Republic of Iran	350 women; mean age 33.77 years (SD: 9.77; range: 18–63)	Cross-sectional	Female Sexual Quality of Life Questionnaire, Female Sexual Distress Scale-revised, Dyadic Sexual Communication Scale, Female Sexual Function Index and Emotional Intimacy Questionnaire	Satisfaction with Life Scale
Boyacıoğlu et al., 2023^79^	Türkiye	169 women and 154 men aged 65 years and older	Cross-sectional	Arizona Sexual Experiences scale	General Health Questionnaire-28 and Control, Autonomy, Self-Realization and Pleasure-19
Florkiewicz-Danel et al., 2023^83^	Poland	65 women aged 18–45 years	Cross-sectional	Mell–Krat Questionnaire and Sexual Satisfaction Scale for Women	General Health Questionnaire-28
Gök et al., 2023^87^	Türkiye	976 women; mean age 35.45 years (SD: 8.47; range: 18–49)	Cross-sectional	Sexual Quality of Life Questionnaire	Multidimensional Scale Of Perceived Social Support and Beck Depression Inventory
Karakose et al., 2023^85^	USA	102 couples; mean age 30.06 years (SD: 5.55; range: 21–50)	Dyadic cross-sectional	New Sexual Satisfaction Scale	Depression Anxiety and Stress Scale-21
Pasha et al., 2023^42^	Islamic Republic of Iran	77 women; age not reported	Cross-sectional	Female Sexual Function Index	General Health Questionnaire
Tavares et al., 2023^90^	Portugal	257 couples; mean age women 29.9 years (SD: 4.7); men 31.6 (SD: 4.9)	Dyadic Longitudinal	International index of erectile function; Female Sexual Function Index and Female Sexual Distress Scale-revised	Edinburgh Postnatal Depression Scale and 7-item Anxiety Subscale of the Hospital Anxiety and Depression Scale
Cabo et al., 2024^62^	USA	1033 men aged ≥ 18 years; median age 55 years (IQR: 35–67)	Cross-sectional	International Index of Erectile Function-5 and Premature Ejaculation Diagnostic Tool	EuroQol-5 Dimension, Visual Analogue Scale and Basic Health Literacy Screen
Przydacz et al., 2024^78^	Poland	3001 men^a^ aged ≥ 18 years	Cross-sectional	International Index of Erectile Function-5 and Premature Ejaculation	7-item Anxiety Subscale of the Hospital Anxiety and Depression Scale

aOR: adjusted odds ratio; CI: confidence interval; IQR: interquartile range; OR: odds ratio; SD: standard deviation; SE: standard error; WHO: World Health Organization.

^a^ stratified sampling.

^b^ cluster sampling.

^c^ simple random sampling.

^d^ quota sampling.

^e^ population-based sampling.

Table 3 presents the summary of the outcomes in each article. Based on the identified health indicators, we divided the narrative synthesis into two categories: (i) sexual and physical health and quality of life, comprising studies assessing the interplay between sexual health, quality of life and health-related quality of life; and (ii) sexual and psychological health and subjective well-being, including studies addressing the interplay between sexual health, psychopathology and well-being.

**Table 3:** Outcome summary of the studies included on the systematic review on associations between sexual health and well-being

Study	Country	Outcome results
Tracy & Junginger, 2007^73^	USA	Psychological symptoms were significantly associated (*P* < 0.001) with: decreased arousal (*r*: 0.22); orgasm (*r*: −0.22); satisfaction (*r*: 0.22); overall sexual functioning (*r*: −0.21); and increased difficulty with lubrication during sexual activity (*r*: −0.20)
Davison et al., 2009^93^	Australia	In univariate analysis, being sexually dissatisfied was associated with lower general psychological well-being (*β*: 4.75; 95% CI: −8.51 to −0.99). Similar results in a multivariate analysis (*β*: = 4.73; 95% CI: −8.48 to −0.97)
Rosen et al., 2009^37^	USA	The odds of sexual distress were elevated for respondents with low desire and self-reported current depression (using antidepressants OR: 1.53; 95% CI: 1.32 to 1.77 or without antidepressant use OR: 1.91; 95% CI: 1.62 to 2.24), with an Short Form Survey social functioning score in the two lowest categories (scores 1–40 OR: 2.00; 95% CI:1.75 to 2.29; and scores 41–50 OR: 1.73; 95% CI:1.51 to 1.98) and with a history of anxiety (OR: 1.61; 95% CI: 1.40 to 1.85)
Sangi-Haghpeyka et al., 2009^40^	USA	Among the female residents, high levels of stress were associated with overall sexual dysfunction (aOR: 3.54; 95% CI: 1.52 to 8.87), low desire (aOR: 2.57; 95% CI: 1.14 to 5.93), arousal problems (aOR: 3.1; 95% CI: 1.28 to 8.44), feeling dissatisfied with sexual life (aOR: 3.92; 95% CI: 1.64 to 10.23). Among the male residents, high levels of stress increased odds for being dissatisfied with sexual life (aOR: 4.94; 95% CI: 2.26 to 11.43) and overall sexual dysfunction (aOR: 7.91; 95% CI: 2.1 to 52.21). All residents who had sexual dysfunction and were dissatisfied with their sex life had significantly lower scores (and percentiles) on quality of life compared with those without any sexual problems and were sexually satisfied (*P* < 0.05)
Çaliskan et al., 2010^33^	Türkiye	Sexual function correlated with well-being scores
Holmberg et al., 2010^64^	Canada	In the same-sex relationship group, the index score correlated negatively (*P* < 0.001) with the State–Trait Anxiety Inventory (*r*: −0.44), depression (*r*: −0.40) and stress (*r*: −0.33) scores. In the mixed-relationship group, the index and inventory scores correlated negatively (*P* < 0.001 and *P* < 0.005, respectively) with the anxiety (index *r*: −0.34 and inventory *r*: −0.23), stress (index *r*: −0.29 and inventory *r*: −0.21) and depression (index *r*: −0.38 and inventory *r*: −0.23) scores. The index scores were also correlated with physical symptoms (*r*: −0.23; *P* < 0.001) and general health (*r*: 0.22; *P* < 0.005). Better sexual satisfaction predicted fewer mental health problems for women in same-sex relationships (*β*: −0.43, *P* < 0.01) and for women in mixed-sex relationships (*β*: −0.44; *P* < 0.001). Better sexual satisfaction was also a moderately strong predictor of fewer physical health difficulties (*β*: −0.33; *P* < 0.01) in the mixed-sex relationship group
Smith et al., 2010^67^	USA	Presenting mild or severe erectile difficulties was associated with reporting depressive symptoms (OR: 2.9; 95% CI:1.71 to 4.91; and OR: 9.3; 95% CI: 3.72 to 23.1, respectively)
Chao et al., 2011^61^	China, Taiwan	The model tested the relationships among latent variables of sexual desire and satisfaction. The verification of each dimension indicated an influence of sexual satisfaction on quality of life: sexual desire to sexual satisfaction (PCE: 0.59; *P* < 0.001), and sexual satisfaction to quality of life (PCE: 0.53; *P* < 0.001). Sexual desire has an indirect coefficient effect on quality of life of 0.313
Shindel et al., 2011^68^	USA	Higher levels of sexual function were linked to fewer depressive symptoms (OR: 0.83; 95% CI: 0.78 to 0.88)
Chang et al., 2012^74^	China, Taiwan	Depression symptoms in early pregnancy were significant negative predictors of overall sexual function (*β*: −0.51; *P* < 0.001), arousal (*β*: −0.08, *P*: 0.01), lubrication (*β*: −0.13; *P*: 0.002), orgasm (*β*: 0.08, *P*: 0.002) and pain (*β*: −0.13; *P* < 0.001). Depressive symptoms in late pregnancy were significant negative predictors of sexual desire (*β*: −0.03, *P* < 0.001) and satisfaction (*β*: −0.06; *P* < 0.001)
Ferreira et al., 2012^38^	Brazil	Sexual quotient final score was associated with perceived quality of life (*P*: 0.042). A sexual quotient final score of bad to poor was associated with poor quality of life (*P*: 0.002)
Dogan et al., 2013^94^	Türkiye	Sexual quality of life was positively correlated to happiness (*r*: 0.42; *P* < 0.001) and satisfaction with life (*r*: 0.5, *P* < 0.001). The model showed that sexual quality of life was a significant positive predictor of happiness (*β*: 0.44; *P* < 0.001) and satisfaction with life (*β*: 0.50; *P* < 0.001)
Nik-Azin et al., 2013^35^	Islamic Republic of Iran	Female sexual function had a significant negative weak correlation with anxiety (*P* < 0.05) and depression(*P* < 0.01). Significant positive weak correlations between female sexual function and general quality of life, psychological health and environment dimensions were found. Only the depression scores predicted female sexual function significantly (*β*: −0.22; *P* < 0.01). Results showed that depression predicted a significant proportion of variance of female sexual function; although weak (*R^2^*: 0.043; *P* < 0.01)
Pastuszak et al., 2013^77^	USA	Significant negative correlations were observed between the total patient questionnaire score and several domains of the erectile function index, including sexual desire (*r*: 0.21; *P*: 0.006), intercourse satisfaction (*r*: 0.29; *P* < 0.001) and overall satisfaction (*r*: 0.413; *P* < 0.001). Each individual question of the sexual desire, intercourse satisfaction and overall satisfaction domains, and one question of the erectile function domain of the erectile function index, showed significant negative correlations with the patient questionnaire score (*P* < 0.05)
Ribeiro et al., 2014^72^	Brazil	Women with sexual dysfunction had significantly higher mean total scores on the depression test than women without sexual dysfunction symptoms (14.2+8.9 versus 8.5+6.0, respectively; *P* < 0.001). Depression (Beck Depression Inventory scores > 21) was seven times higher in pregnant women with sexual dysfunction symptoms than women without sexual dysfunction symptoms (21% versus 3%, respectively; *P* < 0.001)
Flynn & Gow, 2015^55^	United Kingdom	Frequency and importance of sexual behaviours were positively correlated with quality of life (*r*: 0.52 and 0.47, respectively; *P* < 0.001). Sexual frequency was significantly associated with the social relationships domain of the WHO survey (*β*: 0.225; *P* < 0.05). Importance of sexual behaviours was a significant predictor of the psychological domain of the WHO survey (*β*: 0.151; *P*: 0.047)
Kim & Kang, 2015^56^	Republic of Korea	The degree of depression differed significantly based on the frequency of sexual intercourse with the spouse (*F*: 9.92; *P* < 0.001). Individuals with more severe depression had lower intercourse frequency. Quality of life differed significantly according to frequency of intercourse (*F*: 5.76; *P*: 0.001). Sexual quality of life predicted quality of life (*β*: 0.11; *P*: 0.021)
del Mar Sánchez-Fuentes & Sierra, 2015^65^	Spain	Sexual satisfaction was negatively correlated with psychopathological symptoms for heterosexuals (*r*: −0.28; *P* < 0.01) and for homosexuals (*r*: −0.24; *P* < 0.01) and positively correlated with better physical health for heterosexuals (*r*: 0.21; *P* < 0.01). In heterosexual individuals, sexual satisfaction was predicted by vitality (*β*: 0.05; *P* < 0.05) and depression (*β*: −0.06; *P* < 0.05; *F*: 230.92; *P* < 0.001; *R^2^* ; 0.32)
Ghazanfarpour et al., 2016^50^	Islamic Republic of Iran	Women with menopausal symptoms had more sexual problems than women without those symptoms: hot flashes (*P*: 0.01), headache and neck pains (*P*: 0.03), reduced physical strength (*P*: 0.02), weight gain (*P*: 0.01) and pain or leg cramps (*P*: 0.03)
Muise et al., 2016^84^	Canada	Sexual frequency had a positive linear association with satisfaction with life, (*β*: 0.16; *P*: 0.02) and a significant curvilinear association (*β*: −0.15; *P*: 0.03). There was a significant indirect curvilinear effect of sexual frequency on life satisfaction through relationship satisfaction (95% CI: −0.09 to −0.02). However, when relationship satisfaction was included in the model (*β*: 0.51; *P* < 0.001) both the linear and curvilinear associations between sexual frequency and well-being did not reach statistical significance
Schlichthorst et al., 2016^63^	Australia	Sexual difficulties (lack of interest, enjoyment, feeling anxious during sex, not reaching climax or reaching too quickly, and erection difficulties) were linked to self-rated health scores in the well-being survey in both 18–34 and 35–55 age groups (*P* < 0.05)
Alidost et al., 2017^53^	Islamic Republic of Iran	Quality of life and age directly correlated with sexual dysfunction, while prenatal anxiety and income were indirectly correlated with sexual dysfunction through quality of life (*P* < 0.01)
Debrot et al., 2017^92^ (study 1)	USA	Higher sexual frequency was associated with higher life satisfaction (*β*: 0.26; 95% CI: 0.15 to 0.35) and more frequent affectionate touch (*β*: 0.55; 95% CI: 0.56 to 0.79). Affectionate touch frequency was associated with greater life satisfaction (*β*: 0.30; 95% CI: 0.16 to 0.32). Even though reduced, there was a significant indirect effect of sexual frequency on life satisfaction through affectionate touch frequency (*β*: 0.14; 95% CI: 0.01 to 0.26)
Ellouze et al., 2017^46^	Tunisia	The pain dimension of the Female Sexual Function Index correlated negatively with the depression score, while the satisfaction domain correlated positively with depression (*P* < 0.05). Sexual satisfaction was also associated with the mental component of the well-being survey (*P* < 0.05)
Nazarpour et al., 2017^39^	Islamic Republic of Iran	Female Sexual Function Index total score correlated positively with the WHO survey total score (*r*: 0.29; *P* < 0.001). The multiple linear regression analysis showed that the total sexual function score was a predictive factor of the total score of quality of life (*β*: 0.395; *P* < 0.001)
Wåhlin-Jacobsen et al., 2017^89^	Denmark	Women who did not use combined hormonal contraceptives, reporting mild depressive symptoms, were at a significantly increased risk of impaired sexual function (OR: 12.8 to 25.3; *P* < 0.01), sexual distress (OR: 5.0 to 6.1; *P* < 0.01), low sexual desire (OR: 6.5 to 9.2; *P* < 0.01), and hypoactive sexual desire disorder (OR: 7.3 to 10.0; *P* < 0.001)
Worsley et al., 2017^69^	Australia	Severe depressive symptoms were associated with low sexual desire (OR: 1.88; 95% CI: 1.34 to 2.62)
Abedi et al., 2018^66^	Islamic Republic of Iran	All aspects of sexual function and different domains of health-promoting lifestyle were significantly correlated (*P* < 0.001), except for pain and physical activity. Sexual arousal had the strongest correlation with self-actualization (*r*: 0.56) while pain had the lowest correlation with stress management (*r*: 0.07). Women who had better self-actualization were more likely to have better sexual function than other women (OR: 1.10; 95% CI: 1.06 to 1.14). Women who had a higher health responsibility score were more likely to have better sexual function (OR: 1.06; 95% CI: 1.03 to 1.10). Other variables like interpersonal relations and stress management also correlated with sexual function
Nimbi et al., 2018^75^	Italy	Depression was a significant negative predictor of male sexual desire (*β*: −0.39; SE: 0.42; *P* < 0.003)
Rezaei et al., 2018^44^	Islamic Republic of Iran	Women with higher sexual function scores had significantly higher quality of life in all subscales of Short Form-36 (*P* < 0.05). Physical and mental health were positively correlated with all Female Sexual Function Index domains in postpartum women (*r*: 0.10 to 0.312; *P* < 0.05), except between pain and general and physical health, and desire and physical function. Physical and mental quality of life were predicted by the total scores of Female Sexual Function Index (OR: 0.49; 95% CI: 0.24 to 0.71 and OR: 0.350; 95% CI: 0.2 to 0.62, respectively)
Chang et al., 2019^45^	China, Taiwan	The physical and mental components summary of health-related quality of life were predicted by the total score of the Female Sexual Function Index (*β*: 0.17; 95% CI: 0.12 to 0.22 and *β*: 0.16; 95% CI: 0.10 to 0.22, respectively)
Eleuteri et al., 2019^59^	Italy	Erectile function scores predicted the quality of life scores. Additional analyses demonstrated that each score in the erectile function index contributed to an increase of 0.13 in quality of life score
Jackson et al., 2019^80^	United Kingdom	Declines in sexual frequency were associated with more depressive symptoms (*P* < 0.001) and poorer quality of life (*P* < 0.001) in both sexes, and with lower satisfaction with life in women only (*P* < 0.001). The associations between the declines in sexual frequency and life satisfaction in men differed by age (*P*: 0.037), particularly in those aged 60–69 years (*P*: 0.019)
Meira et al., 2019^36^	Brazil	Women without sexual dysfunction had significantly higher scores on the physical domain and environment (3.6; SD: 0.41; *P*: 0.02 and 3.37; SD: 0.33; *P*: 0.05, respectively) than women with sexual dysfunction (3.09; SD: 0.67 and 2.84; SD: 0.40, respectively)
Oh & Kim, 2019^47^	Republic of Korea	Health-related quality of life was a determinant of sexual function during pregnancy (*β*: 0.18; *P*: 0.03)
Peixoto et al., 2019^51^	Brazil	Sexual desire showed a positive correlation with the Short Form-36 dimensions of vitality (*r*: 0.46; *P*: 0.004) and social aspects (*r*: 0.51; *P*: 0.001), general health status (*r*: 0.35; *P*: 0.03) and mental health (*r*: 0.38; *P*: 0.02). Arousal, orgasm and satisfaction with sexual life presented moderate positive relationships with pain (*r*: 0.40, *P*: 0.01; *r*: 0.42, *P*: 0.01; and *r*: 0.43. *P*: 0.009; respectively). Female Sexual Function Index total score was positively related to pain (*r*: 0.37; *P*: 0.02). Satisfaction with sexual life was positively related to vitality (*r*: 0.33; *P*: 0.04)
Fagundes Ferreira et al., 2020^43^	Brazil	Women with sexual dysfunction had statistically significantly lower general health (42.05; SD: 13.22) than those without sexual dysfunction (50.03; SD: 11.43; *P* < 0.001). Female Sexual Function Index correlated positively with all domains of Short Form-36 (e.g. general health *r*: 0.31; *P* < 0.05). All correlations were weak, except for vitality (*r*: 0.42; *P* < 0.05)
Lu et al., 2020^60^	China	Individuals with no decline in sexual activity had fewer anxiety (6.98; SD: 4.59), depressive symptoms (8.59; SD: 5.62) and higher life satisfaction (42.37; SD: 8.76), compared with individuals that declined sexual frequency (9.86; SD: 5.47; 11.71; SD: 5.53 and 39.80; SD: 9.53; *P* < 0.001; respectively)
Najimi et al., 2020^57^	Islamic Republic of Iran	There was a positive association between sexual quality of life and general health in older men (*r*: −0.41; *P* < 0.001)
NeJhaddadgar et al., 2020^41^	Islamic Republic of Iran	Sexual functioning was positively correlated with health status (*r*: 0.264; *P* < 0.001)
Bigizadeh et al., 2021^34^	Islamic Republic of Iran	Women with normal sexual function had higher levels of physical (*P* < 0.001), psychological (*P* < 0.01), environmental health (*P* < 0.05) and social quality of life (*P* < 0.01), and a greater total quality of life score (*P* < 0.001) than women with sexual dysfunction. The total score of sexual function was highly correlated with the physical dimension of quality of life (*r*: 0.60, *P* < 0.001)
Effati-Daryani et al., 2021^71^	Islamic Republic of Iran	There was a significant negative correlation between the total sexual function score and stress (*r*: −0.203; *P* < 0.001), anxiety (*r*: −0.166; *P*: 0.001) and depression (*r*: −0.234; *P* < 0.001). The general linear model indicated mild anxiety to be a significant negative predictor of sexual function (adjusted *β*: −3.32; 95% CI: −5.70 to −0.94)
Fasero et al., 2021^49^	Spain	Cervantes-SF correlated positive with female sexual function (*ρ*: 0.223; *P* < 0.001). The final logistic regression model identified the use of vaginal hormonal treatment as an independent factor related to sexual function score (*β*exp: 1.759; 95% CI: 1.05 to 2.96)
Jalali et al., 2021^48^	Islamic Republic of Iran	The total scores of sexual self-efficacy measure and menopause-specific quality of life were correlated (*r*: 0.31; *P* < 0.001). Most dimensions of the menopause-specific quality of life were significantly correlated to the sexual self-efficacy dimensions, with the exception of the vasomotor dimension. Sexual desire was a significant predictor of Menopause-Specific Quality of Life 's score (*β*: 0.20; *P* < 0.001)
Mollaioli et al., 2021^82^	Italy	Higher General Anxiety Disorder and patient questionnaire scores were presented by participants reporting no sexual activity during COVID-19 movement restrictions (*β*: 0.89; SE: 0.39; *P* < 0.05; *β*: 0.94; SE: 0.45; *P* < 0.05; respectively). Sexually active participants had a significantly lower risk of developing anxiety and depression than those who were not sexually active during the movement restrictions (OR: 1.32; 95% CI: 1.12 to 1.57 and OR: 1.34; 95% CI: 1.15 to 1.57, respectively). Psychological distress had a direct negative effect on sexual health (sexual well-being indices; *β*: −0.23; *P* < 0.0001 in men and *β* : −0.21; *P* < 0.001 in women). Frequency of sexual activity was a protective mediator between psychological distress (*β*: −0.18; *P* < 0.001 in men and *β*: −0.14; *P* < 0.001 in women) and sexual health (*β*: 0.43; *P* < 0.001 in men and *β*: 0.31; *P* < 0.001 in women)
Soler et al., 2021^70^	Spain	Anxiety negatively predicted men's desire (*β*: −0.16; *P* < 0.001) and arousal (*β*: −0.22; *P* < 0.001). Depression negatively predicted men's erection (*β*: −0.16; *P* < 0.01) and satisfaction (*β*: −0.17; *P* < 0.01); and women's desire (*β*: −0.23; *P* < 0.001) and arousal (*β*: −0.26; *P* < 0.001). Somatization had a negative association (*β*: −0.12; *P* < 0.05) with men's desire
Vedovo et al., 2021^52^	Italy	Overall sexual function correlated with depression symptoms in both trans (*r*: 0.53; *P* ≤ 0.001) and cisgender women (*r*: −0.47; *P* ≤ 0.01). The mental component of quality of life for both trans (*r*: −0.71; *P* ≤ 0.001) and cis women (*r*: 0.57; *P* ≤ 0.001) also correlated with sexual function. The physical component of quality of life only correlated with sexual function in transgender women (*r*: −0.31; *P* ≤ 0.05). The multiple regression analysis showed that the dissatisfaction dimension from the operated Male to Female Sexual Function Index scale contributed to the estimation of the mental component of quality of life in transgender women (*β*:−0.29; *P* ≤ 0.05), while sexual desire emerged in cisgender women (*β*: 0.35; *P* ≤ 0.05)
Chatterjee et al., 2022^54^	India	Erection and lubrication function was predicted by depression (*β*: 0.19; 95% CI: 0.06 to 0.32) and the presence of comorbidities (*β*: 0.53; 95% CI: 0.22 to 0.84). Depression predicted problems in orgasm (*β*: 0.45; 95% CI: 0.19 to 0.71). Depression (*β*: 0.58; 95% CI: 0.35 to 0.81) and anxiety (*β*: 0.28; 95% CI: 0.09 to 0.47) predicted less orgasmic satisfaction. Overall sexual dysfunction was predicted by depression (*β*: 0.3; 95% CI: 0.14 to 0.46
Gil-Salmerón et al., 2022^81^	Spain	Participants with higher levels of depression were associated with significantly lower sexual activity in the fully adjusted model (OR: 0.09; 95% CI: 0.01–0.61). Mild anxiety level was associated with lower sexual activity (OR: 0.40; 95% CI: 0.19 to 0.84). Only four participants had severe anxiety and were excluded from the analysis
Khorshidi et al., 2022^86^	Islamic Republic of Iran	Monthly frequency of sexual intercourse (*r*: 0.26; *P* < 0.001), sexual distress (*r*: −0.61; *P* < 0.001) and psychological distress (*r*: −0.44; *P* < 0.001) were significantly associated with women’s sexual quality of life. Psychological distress (*β*: −0.42; *P* < 0.001), monthly frequency of sexual intercourse (*β*: 0.20; *P* < 0.001) and sexual distress (*β*: −0.14; *P* < 0.001) were significant predictors of women’s sexual quality of life
Oveisi et al., 2022^88^	Canada	Sexual quality of life was a significant positive predictor of mental well-being and self-perceived health status, with each one-unit increase in sexual quality of life associated with a 0.35 increase in mental well-being (95% CI: 0.105 to 0.428)
Pollard; 2022^95^	USA	Depressive symptoms were negatively correlated with sexual satisfaction (*r*: −0.13; *P* < 0.05)
Ryu et al., 2022^76^	Republic of Korea	Quality of life was negative correlated with depression (*r*: −0.51; *P* < 0.001), while self-efficacy (*r*: 0.52; *P* < 0.001) and sexual function (*r*: 0.35; *P* < 0.001) showed a positive correlation. Depression negatively correlated with self-efficacy (*r*: −0.31; *P* < 0.001) and sexual function (*r*: −0.30; *P* < 0.001). Self-efficacy was positively correlated with sexual function (*r*: 0.27; *P* < 0.001)
de Souza Júnior et al., 2022^58^	Brazil	General sexual functioning correlated positively with general quality of life (*ρ*: 0.325; *P* < 0.001)
Bahrami et al., 2023^91^	Islamic Republic of Iran	Sexual functioning was the strongest predictor of life satisfaction among Iranian married women(*β*: 0.17; *P*: 0.009)
Boyacıoğlu et al., 2023^79^	Türkiye	Sexual experiences moderately correlated positively with the general health scores (*r*: 0.327) and negatively with the control, autonomy, self-realization and pleasure scores (*r*: 0.77). Participants without a partner, sexual activity or feelings of sexual attractiveness seemed to experience more sexual dysfunction and psychological problems, and lower quality of life. Older people with sexual dysfunction presented lower general health scores and lower quality of life levels
Florkiewicz-Danel et al., 2023^83^	Poland	There were no significant associations between the frequency of sexual intercourse, sexual functioning, satisfaction and mental health
Gök et al., 2023^87^	Türkiye	Women who used a traditional family planning method, had an unintended pregnancy, an abortion or more than two pregnancies, low levels of social support and depressive symptoms had significantly lower quality of sexual life (*P* < 0.05). The quality of sexual life correlated positively with depression (*r*: 0.416; *P* < 0.001) and social support (total score *r*: 0.373; *P* < 0.001; family subscale *r*: 0.417; *P* < 0.001; and friends subscale *r*: 0.324; *P* < 0.001). The presence of sexual problems (OR: 2.72; 95% CI: 1.51 to 4.88) and social support (OR: 3.65; 95% CI: 2.45 to 5.43) were unique predictors of sexual quality of life
Karakose et al., 2023^85^	USA	Wives' sexual satisfaction was predicted by own stress (estimate: −1.27; SE: 0.49; *P* < 0.01) and depression (estimate: −1.26; SE: 0.49; *P* < 0.05) and husbands' depression (estimate: −0.95; SE: 0.48; *P* < 0.01). Husbands' sexual satisfaction was predicted by own depression (estimate: −1.88; SE: 0.40; *P* < 0.001), anxiety (estimate: −1.57; SE: 0.49; *P* < 0.001) and stress (estimate: −1.57; SE: 0.38; *P* < 0.001)
Pasha et al., 2023^42^	Islamic Republic of Iran	Sexual function score correlated inversely with mental health (*ρ*: −0.430; *P* < 0.001), physical complications (*ρ*: −0.394, *P* < 0.0001), anxiety and insomnia (*ρ*: −0.314; *P* < 0.001), social dysfunction (*ρ*: = −0.262; *P* < 0.004) and depression (*ρ*: −0.409; *P* < 0.001). The findings on the predictors of sexual health on the mental health of married women showed a significant inverse association between sexual health and mental health and its dimensions (*P* < 0.05). The linear regression analysis showed that the variables of sexual health (*β*: −0.430; *P* < 0.001) were predictors of mental health. Sexual health factors explained 18.5% of mental health variance
Tavares et al., 2023^90^	Portugal	Couples in discrepant sexual function class showed increased levels of anxiety and depression in women at 20 weeks of pregnancy (*χ^2^*: 7.72; *P*: 0.005 and*χ*^2^: 7.61; *P*: 0.006, respectively) and 3 months postpartum (*χ*^2^: 6.87; *P*: 0.009 and *χ*^2^: 14.29; *P* < 0.001, respectively) compared to couples in the high sexual function class. Couples in the low sexual distress class presented lower levels of anxiety and depression at baseline for pregnant women (*χ*^2^: 31.63; *P* < 0.001 and; *χ*^2^: 21.94; *P* < 0.001, respectively) and for fathers (*χ*^2^: 17.69; *P* < 0.001 and *χ*^2^: 15.14; *P* < 0.001, respectively), and at 3 months postpartum (*χ*^2^: 33.14; *P* < 0.001 and *χ*^2^: 15.03, *P* < 0.001, respectively, for mothers, and *χ*^2^: 10.2, *P* < 0.001 and *χ*^2^: 19.4; *P* < 0.001, respectively, for fathers)
Cabo et al., 2024^62^	USA	Higher erectile function and lower premature ejaculation scores, better overall health-related quality of life and having a sexual partner within the last month were associated with an increased likelihood of overall sexual satisfaction. When stratified by age, higher erectile function scores were consistently positively associated with sexual satisfaction (OR: 1.18; 95% CI: 1.15 to 1.22) and independently associated with improved overall health-related quality of life (*β*: 0.71; SE: 0.08; *P* < 0.001)
Przydacz et al., 2024^78^	Poland	Sexual variables were significantly associated with mental health

aOR: adjusted odds ratio; CI: confidence interval; COVID-19: coronavirus disease 2019; OR: odds ratio; PCE: path coefficient estimates; SD: standard deviation; SE: standard error; WHO: World Health Organization.

### Sexual and physical health

Included studies^33^^–^^65^ found statistically significant associations between sexual health and quality of life. Female sexual function was found to be positively correlated with multiple domains of quality of life,^33^ namely psychological,^34^^,^^35^ environmental,^34^^–^^36^ social^34^^,^^37^ and overall quality of life.^34^^,^^35^^,^^38^^–^^40^ Female sexual function was also found to be positively linked to health status,^41^^,^^42^ health-related quality of life^43^^,^^44^ and its physical^34^ and psychological components.^45^^,^^46^ A similar association between female sexual function and health-related quality of life was found during pregnancy.^47^ Sexual function was also linked to menopause-specific quality of life,^48^^,^^49^ with women experiencing declining sexual function reporting more menopausal symptoms.^50^ After menopause, sexual desire was linked to greater vitality, while arousal and orgasm were linked to lower experience of physical pain, possibly due to the effect of sexual hormones on pain perception.^51^ Better overall sexual function in transgender women is associated with higher scores on the psychological and mental components of health-related quality of life.^52^ A study on sexuality and well-being during pregnancy revealed that quality of life might mediate the association between sexual function and prenatal anxiety, showing that higher levels of prenatal anxiety are linked to a decrease in quality of life, which in turn negatively affects sexual function.^53^ However, an inquiry of the general population during the enforcement of movement restrictions due to the coronavirus disease 2019 (COVID-19) pandemic, showed that sexual dysfunction was not statistically associated with quality of life among respondents.^54^

Frequency of sexual activities,^55^ sexual quality of life^56^^,^^57^ and male sexual function^40^^,^^58^ were positively associated with quality of life. Men presenting erectile^59^ and orgasmic difficulties reported lower quality of life.^60^ A structural equation model indicated that increased sexual satisfaction was associated with improved quality of life in older age.^61^ Men with higher scores on overall sexual function also presented higher levels of health-related quality of life.^62^^,^^63^

Positive associations between sexual satisfaction and health-related quality of life were also found.^62^ Women in mixed and same-sex relationships reporting higher levels of sexual satisfaction presented fewer physical symptoms and better health.^64^ Sexual satisfaction in same-sex and mixed-sex relationships correlated positively with the physical dimension of health-related quality of life. Sexual satisfaction was also correlated with vitality.^51^^,^^65^

An analysis of the relationship between sexual function and health-promoting lifestyles showed that health behaviours correlated positively with sexual function domains. However, while physical activity had a positive significant association with all sexual dimensions, it showed a non-significant association with sexual pain.^66^

### Sexual and psychological health 

Despite recent acknowledgement of the holistic nature of sexual health, most studies linking sexual health and mental health focused on sexual function. Higher sexual function levels were linked to fewer psychological symptoms and higher psychological functioning.^67^^–^^70^ Multiple female sexual function domains negatively correlated with anxiety and depression scores.^41^^,^^42^^,^^46^^,^^71^ Women presenting low sexual desire tended to report higher levels of depression,^69^ and sexual dysfunction was also associated with increased depression in pregnant women.^72^ Similar findings were found in women with sexual and gender diversity, whose psychological symptoms were associated with overall sexual function^52^ and specific dimensions of female sexual function, except for sexual desire and pain.^73^ Regarding overall psychological health, a positive correlation was found with overall sexual function^52^ and sexual desire in postmenopausal women.^51^ Female sexual arousal negatively correlated with depressive symptoms and orgasmic function correlated negatively with anxiety and depression.^54^ During early pregnancy, depressive symptoms were negatively associated with overall sexual function, orgasm and pain, while during late pregnancy, it correlated negatively with sexual desire and satisfaction.^74^ Men with sexual desire^75^ and erectile and orgasmic difficulties^60^^,^^76^ reported more psychopathological symptoms than those with unimpaired sexual function. Multiple domains of male sexual function, including overall sexual function,^76^ sexual desire, intercourse satisfaction and overall satisfaction,^77^ correlated negatively with depressive symptoms.

Less frequent sexual activities were associated with reporting more psychological problems in both adults^56^^,^^78^ and older adults.^79^^,^^80^ Also intercourse frequency was associated with mental health indicators, particularly during COVID-19 quarantines.^81^^,^^82^ However, contrasting results indicate no significant associations between intercourse frequency and mental health aspects, such as somatic symptoms, anxiety and depression during early motherhood, raising questions about the impact of frequency on mental health.^83^ Moreover, a curvilinear association between sexual frequency and well-being was found, indicating that increased sexual frequency was associated with higher well-being, but this association was not significant at frequencies greater than once a week.^84^

Sexual satisfaction was inversely linked to psychopathological symptoms in heterosexual and homosexual individuals.^64^^,^^65^ In older women, sexual satisfaction also correlated negatively with depression and anxiety.^41^ A couple study suggested that women's sexual satisfaction was negatively correlated with depression and partner depression scores, while men's sexual satisfaction was negatively correlated with anxiety.^85^

Sexual quality of life was found to be inversely linked to psychological distress,^86^ depression scores^87^ and overall mental health.^88^ Sexual distress was also found to be linked to presenting mild depressive symptoms in premenopausal women.^89^ Similarly, a longitudinal study found that couples with less sexual distress showed reduced symptoms of anxiety and depression.^90^

Included studies also found associations between sexual health and subjective well-being. Among subjective measures, life satisfaction and overall well-being correlated with sexual health. Life satisfaction was positively associated with female sexual function,^91^ and negatively with erectile and orgasm difficulties in men.^60^ More frequent sexual activities, including fondling and caressing, were positively linked with life satisfaction.^79^^,^^80^^,^^92^ However, when considering relationship satisfaction, the association between sexual activity frequency and life satisfaction was not statistically significant.^84^ A positive association between women’s sexual satisfaction and psychological well-being was also shown.^93^ Sexual quality of life correlated positively with happiness and life satisfaction in married women.^94^

## Discussion

This systematic review aimed to clarify the association between sexual health, health and well-being. Using WHO’s positive and multidimensional definition of health,^3^^,^^21^ we analysed the links between physical, emotional and mental health, social well-being, sexual health, and overall well-being.

Overall, the results presented in the included studies confirmed associations between sexual health, health and well-being. Specifically, findings suggest significant positive associations between sexual health, lower levels of depression and/or anxiety, and life satisfaction in both men and women, including older adults, pregnant women, and people in same-sex and mixed-sex relationships. Studies on quality of life and health-related quality of life also consistently showed significant positive associations with sexual health. Female sexual function is positively linked with overall health status, health-related quality of life and specific health dimensions. Men with better erectile and orgasmic function reported higher quality of life, while difficulties in these areas were associated with lower quality of life. These findings were mostly observed in heterosexual samples of men and women, across all age groups.

Various studies showed that sexual satisfaction was a key factor linked to improved quality of life. For example, sexual satisfaction was positively associated with better health status, fewer physical symptoms and higher psychological well-being.^64^^,^^65^ Sexual function, particularly in women, correlates strongly with multiple aspects of quality of life, such as psychological, environmental and social dimensions.^34^^–^^40^ This association extends across life stages, such as pregnancy, menopause and post-menopause,^47^^–^^51^ as well as in older age, where sexual function is indirectly associated with improved quality of life through its influence on mental health.^61^ Notably, evidence exists of a positive association between sexual health and mental health outcomes. Lower levels of anxiety and depression are strongly linked to better sexual function and satisfaction.^41^^–^^43^^,^^52^^,^^60^^,^^71^^,^^76^

We identified contradictory findings regarding the importance of sexual frequency, particularly for mental health and well-being. Although evidence suggests that increased sexual frequency is associated with better health outcomes,^56^^,^^78^^–^^82^ one study found no association between intercourse frequency and several mental health outcomes during early motherhood;^83^ and another study showed a curvilinear association between sexual frequency and well-being, regardless of demographic group.^84^ The latter study^84^ suggests that while sexual frequency may enhance well-being, the benefits might plateau at higher frequencies and may depend on contextual and situational factors that can mediate or disrupt these associations.

While the search strategy encompassed a holistic approach to sexual health,^30^ most studies focused on sexual function, with some studies assessing sexual satisfaction, sexual quality of life, sexual activity or frequency and sexual distress. These findings highlight that sexual health often appears to be conceptualized primarily as the absence of infirmity, despite significant efforts to comprehensively define and address sexual health.^1^^,^^3^

Additionally, issues regarding the operationalization of sexual health, namely sexual function, are reported in the literature^106^ and hinder assessment standardization and research comparability. Similarly, only 17 studies used multiple validated measures for assessing health, revealing a paucity of multidimensional approaches to health as recommended by WHO.^21^^,^^101^ While measures like the Female Sexual Function Index, International Index of Erectile Function-5, and Female Sexual Distress Scale offer important metrics for sexual functioning and distress, these tools may not fully capture the multidimensional nature of sexual health as outlined by WHO, which includes aspects like sexual satisfaction, pleasure, competency and consent.^28^ Consent is fundamental to sexual health, yet few included studies explicitly measured whether sexual activities were consensual, nor did survey instruments routinely assess this factor. Moreover, the critical dimension of sexual pleasure, an essential component of positive sexual health, was often overlooked in assessment tools, limiting their ability to reflect the full experience of sexual well-being. Including consent and pleasure as factors in sexual health assessments could provide a more holistic view of sexual well-being and safety. 

Furthermore, overlapping constructs – for example, sexual pleasure and sexual satisfaction; sexual health and sexual well-being – complicate the study of associations. These challenges result from the scarcity of theoretical frameworks informing measurement and validation in assessments of sexual health outcomes.^107^^,^^108^ The issue is further complicated when studies either fail to specify the meaning of sex, allowing respondents to interpret the term themselves,^109^ or apply inconsistent definitions to sexual activity when studying various sexuality-related outcomes. Therefore, a comprehensive operationalization of sexual health that carefully defines sexual activity, proposing a broad measure to assess the full spectrum of what being sexually healthy entails, is needed.

While many of the included studies were conducted in low- and middle-income countries, we found no research from the African region. This underrepresentation suggests that, despite the substantial positive effect of the reproductive health strategy, research focusing on the positive dimensions of sexual health remains underprioritized, particularly in low- and middle-income countries.^110^ Most studies also included non-representative samples and used cross-sectional analyses, limiting the generalizability of results, and precluding inferences about causal relationships between sexual health, health and well-being. Regarding sample characteristics, although studies included a wide age range covering the lifespan, sexually and gender-diverse minorities were underrepresented. Moreover, results from the risk of bias assessment indicate significant bias concerns. No study in this systematic review demonstrated a complete absence of bias in estimating a potential causal effect of sexual health factors such as sexual function, frequency or satisfaction on health and well-being indicators such as psychological health, quality of life and life satisfaction. Approximately half of the studies showed a high risk of bias due to a lack of control or adjustment for key confounding factors. Contextualization is critical for accounting for confounding effects when explaining the link between sexual activity and health.^17^ The need for contextualization applies to the associations between sexual health and overall health and well-being. Sexual health encompasses various biological, psychological and social processes that might influence health or moderate the associations between these domains. For example, chronic diseases, socioemotional adjustment, being in a (consensual) relationship, or access to sexual and reproductive health-care services can all affect sexual health. A comprehensive conceptualization is mandatory for assessing the interplay between sexual health, health and well-being, as well as investigating the causality of these associations. 

This review has some limitations. The first limitation is the exclusion of some dimensions of sexual health, as preventing gender-based violence and sexually transmissible infections are equally integral to being sexually healthy.^30^ Second, the use of specific search terms directed at sexual health, health and well-being might have constrained the search results. Finally, methodological issues such as the initial screening by title should be acknowledged.

Our findings indicate that a positive approach to sexual health may play an important role in improving health and well-being, aligning with the recent emphasis by WHO^111^ and the World Association for Sexual Health^112^ on the positive dimensions of sexual health as central to advancing sexual health and rights. However, research on sexual health and its relationship with overall health and well-being remains limited, and more robust research in this area is needed. A more holistic view of sexual health, encompassing not only functioning and distress but also satisfaction, pleasure, consent and broader psychosocial aspects, could inform more effective health promotion strategies in the future.

## References

[1] Defining sexual health: report of a technical consultation on sexual health, 28–31 January 2002. Geneva: World Health Organization; 2006. Available from: https://www.cesas.lu/perch/resources/whodefiningsexualhealth.pdf [cited 2024 Jul 15].

[2] Resolution A/RES/70/1. Transforming our world: the 2030 agenda for sustainable development. In: Seventieth United Nations General Assembly, New York, 25 September 2015. New York: United Nations; 2015. Available from: https://sdgs.un.org/2030agenda [cited 2024 Jul 15].

[3] Sexual health, human rights and the law. Geneva: World Health Organization; 2015. Available from: https://www.who.int/publications/i/item/9789241564984 [cited 2024 Jul 15].

[4] WAS declaration on sexual pleasure [internet]. Stellenbosch: World Association for Sexual Health; 2019. Available from: https://www.worldsexualhealth.net/was-declaration-on-sexual-pleasure [cited 2024 Jul 15].

[5] Sladden T, Philpott A, Braeken D, Castellanos-Usigli A, Yadav V, Christie E, et al. Sexual health and wellbeing through the life course: ensuring sexual health, rights and pleasure for all. Int J Sex Health. 2021 Nov 5;33(4):565–71. 10.1080/19317611.2021.1991071 38595782 PMC10903615

[6] Katz A. Sexuality and illness: a guidebook for health professionals. London: Routledge; 2021. 10.4324/9781003145745 10.4324/9781003145745

[7] Gianotten WL. The health benefits of sexual expression. In: Geuens S, Polona Mivšek A, Gianotten WL, editors. Midwifery and sexuality. Cham: Springer International Publishing; 2023. pp. 41–8. 10.1007/978-3-031-18432-1_4

[8] Liu H, Waite LJ, Shen S, Wang DH. Is sex good for your health? A national study on partnered sexuality and cardiovascular risk among older men and women. J Health Soc Behav. 2016 Sep;57(3):276–96. 10.1177/0022146516661597 27601406 PMC5052677

[9] De Berardis G, Franciosi M, Belfiglio M, Di Nardo B, Greenfield S, Kaplan SH, et al.; Quality of Care and Outcomes in Type 2 Diabetes (QuED) Study Group. Erectile dysfunction and quality of life in type 2 diabetic patients: a serious problem too often overlooked. Diabetes Care. 2002 Feb;25(2):284–91. 10.2337/diacare.25.2.284 11815497

[10] Brody S. The relative health benefits of different sexual activities. J Sex Med. 2010 Apr;7(4 Part 1):1336–61. 10.1111/j.1743-6109.2009.01677.x 20088868

[11] Rosen RC, Bachmann GA. Sexual well-being, happiness, and satisfaction, in women: the case for a new conceptual paradigm. J Sex Marital Ther. 2008;34(4):291–307. 10.1080/00926230802096234 18576229

[12] Vasconcelos P, Paúl C, Serruya SJ, Ponce de León RG, Nobre P. A systematic review of sexual health and subjective well-being in older age groups. Rev Panam Salud Publica. 2022 Oct 25;46:e179. 10.26633/RPSP.2022.179 36320206 PMC9595221

[13] Allen MS. Sexual activity and cognitive decline in older adults. Arch Sex Behav. 2018 Aug;47(6):1711–9. 10.1007/s10508-018-1193-8 29767822

[14] Hsu B, Hirani V, Waite LM, Naganathan V, Blyth FM, Le Couteur DG, et al. Temporal associations between sexual function and cognitive function in community-dwelling older men: the Concord Health and Ageing in Men Project. Age Ageing. 2018 Nov 1;47(6):900–4. 10.1093/ageing/afy088 29893766

[15] Smith L, Grabovac I, Yang L, López-Sánchez GF, Firth J, Pizzol D, et al. Sexual activity and cognitive decline in older age: a prospective cohort study. Aging Clin Exp Res. 2020 Jan;32(1):85–91. 10.1007/s40520-019-01334-z 31494914

[16] Graugaard C. Sexuality as a health-promoting factor - theoretical and clinical considerations. Nat Rev Urol. 2017 Oct;14(10):577–8. 10.1038/nrurol.2017.117 28720864

[17] Diamond LM, Huebner DM. Is good sex good for you? Rethinking sexuality and health. Soc Personal Psychol Compass. 2012;6(1):54–69. 10.1111/j.1751-9004.2011.00408.x

[18] Sexual and reproductive health and rights: an essential element of universal health coverage. New York: United Nations Population Fund; 2019. Available from: https://www.unfpa.org/sites/default/files/pub-pdf/SRHR_an_essential_element_of_UHC_2020_online.pdf [cited 2024 Jul 15].

[19] Page MJ, McKenzie JE, Bossuyt PM, Boutron I, Hoffmann TC, Mulrow CD, et al. The PRISMA 2020 statement: an updated guideline for reporting systematic reviews. BMJ. 2021 Mar 29;372(71):n71. 10.1136/bmj.n71 33782057 PMC8005924

[20] Vasconcelos P, Carrito M, Quinta-Gomes AL, Patrão AL, Nobre P. Is sexual health associated with overall health and well-being? Findings from a systematic review. [Prospero registration]. York: University of York Centre for Reviews and Dissemination; 2024. Available from: https://www.crd.york.ac.uk/prospero/display_record.php?ID=CRD42024507701 [cited 2024 Oct 30].

[21] Summary report on proceedings minutes and final acts of the International Health Conference held in New York from 19 June to 22 July 1946. New York: United Nations, World Health Organization, Interim Commission; 1948. Available from: https://iris.who.int/handle/10665/85573 [cited 2024 Jul 15].

[22] Felce D, Perry J. Quality of life: its definition and measurement. Res Dev Disabil. 1995 Jan-Feb;16(1):51–74. 10.1016/0891-4222(94)00028-8 7701092

[23] Skevington SM, Lotfy M, O’Connell KA; WHOQOL Group. The World Health Organization’s WHOQOL-BREF quality of life assessment: psychometric properties and results of the international field trial. A report from the WHOQOL group. Qual Life Res. 2004 Mar;13(2):299–310. 10.1023/B:QURE.0000018486.91360.00 15085902

[24] Stephenson KR, Meston CM. Differentiating components of sexual well-being in women: are sexual satisfaction and sexual distress independent constructs? J Sex Med. 2010 Jul;7(7):2458–68. 10.1111/j.1743-6109.2010.01836.x 20456625

[25] Heiman JR, Long JS, Smith SN, Fisher WA, Sand MS, Rosen RC. Sexual satisfaction and relationship happiness in midlife and older couples in five countries. Arch Sex Behav. 2011 Aug;40(4):741–53. 10.1007/s10508-010-9703-3 21267644

[26] Fielder R. Sexual Functioning. In: Gellman MD, Turner JR, editors. Encyclopedia of behavioral medicine. New York: Springer; 2013. pp. 1774–7. 10.1007/978-1-4419-1005-9_668

[27] Lawrance KA, Byers ES. Sexual satisfaction in long-term heterosexual relationships: the interpersonal exchange model of sexual satisfaction. Pers Relatsh. 1995;2(4):267–85. 10.1111/j.1475-6811.1995.tb00092.x

[28] Measuring sexual health: conceptual and practical considerations and related indicators. Geneva: World Health Organization; 2010. Available from: https://iris.who.int/handle/10665/70434 [cited 2024 Jul 15].

[29] Health promotion glossary of terms 2021. Geneva: World Health Organization; 2021. Available from: https://www.who.int/publications/i/item/9789240038349 [cited 2024 Oct 16].

[30] Sexual health and its linkages to reproductive health: an operational approach. Geneva: World Health Organization; 2017. Available from: https://iris.who.int/bitstream/handle/10665/258738/9789241512886-eng.pdf?sequence=1 [cited 2024 Jul 15].

[31] Higgins JPT, Morgan RL, Rooney AA, Taylor KW, Thayer KA, Silva RA, et al. A tool to assess risk of bias in non-randomized follow-up studies of exposure effects (ROBINS-E). Environ Int. 2024 Apr;186:108602. 10.1016/j.envint.2024.108602 38555664 PMC11098530

[32] McGuinness LA, Higgins JPT. Risk-of-bias VISualization (robvis): an R package and Shiny web app for visualizing risk-of-bias assessments. Res Synth Methods. 2021 Jan;12(1):55–61. 10.1002/jrsm.1411 32336025

[33] Çaliskan E, Çorakçi A, Doger E, Coskun E, Özeren S, Çorapçioglu A. Evaluation of sexual function and quality of life in menopausal transition and menopause in a cohort of Turkish women. Turk Klin Tip Bilim Derg. 2010;30(5):1517–23. 10.5336/medsci.2008-9799

[34] Bigizadeh S, Sharifi N, Javadpour S, Jamali S. Sexual function and quality of life in pregnant Iranian women. Sex Relationship Ther. 2021;36(2-3):276–84. 10.1080/14681994.2020.1787372

[35] Nik-Azin A, Nainian MR, Zamani M, Bavojdan MR, Bavojdan MR, Motlagh MJ. Evaluation of sexual function, quality of life, and mental and physical health in pregnant women. J Family Reprod Health. 2013 Dec;7(4):171–6. 24971121 PMC4064754

[36] Meira LF, De Morais KCS, De Sousa NA, Ferreira JB. Função sexual e qualidade de vida em mulheres climatéricas. Fisioter Bras. 2019;20(1):101–8. Portuguese. 10.33233/fb.v20i1.2672

[37] Rosen RC, Shifren JL, Monz BU, Odom DM, Russo PA, Johannes CB. Correlates of sexually related personal distress in women with low sexual desire. J Sex Med. 2009 Jun;6(6):1549–60. 10.1111/j.1743-6109.2009.01252.x 19473457

[38] Ferreira DQ, Nakamura MU, Souza E, Mariani Neto C, Ribeiro MC, Santana T, et al. [Sexual function and quality of life of low-risk pregnant women]. Rev Bras Ginecol Obstet. 2012 Sep;34(9):409–13. Portuguese. 10.1590/S0100-72032012000900004 23197279

[39] Nazarpour S, Simbar M, Ramezani Tehrani F, Alavi Majd H. Quality of life and sexual function in postmenopausal women. J Women Aging. 2018 Jul-Aug;30(4):299–309. 10.1080/08952841.2017.1395539 29077541

[40] Sangi-Haghpeykar H, Ambani DS, Carson SA. Stress, workload, sexual well-being and quality of life among physician residents in training. Int J Clin Pract. 2009 Mar;63(3):462–7. 10.1111/j.1742-1241.2008.01845.x 19222631

[41] NeJhaddadgar N, Ziapour A, Abbas J, Mardi A, Zare M. Correlation between general health and sexual function in older women in an Iranian setting. J Educ Health Promot. 2020 Nov 26;9(1):300. 10.4103/jehp.jehp_316_20 33426104 PMC7774623

[42] Pasha H, Khalajinia Z, Yadollahpour MH, Gholinia H. Sexual function, religion, existential well-being and mental health among Iranian married women of reproductive age. J Relig Health. 2023 Oct;62(5):3399–413. 10.1007/s10943-023-01835-2 37226017

[43] Fagundes Ferreira F, La Rosa VL, Nepomuceno Benites M, Marques Cerentini T, Machado de Souza C, Caruso S, et al. Sexual function evaluation in Brazilian women accessing a public health service: an observational cross-sectional study. Sexual and Relationship Therapy. 2020; 38(2):219–29. 10.1080/14681994.2020.1817365

[44] Rezaei N, Janani F, Sharifi N, Omidi F, Azadi A. Sexual function and quality of life among postpartum women: a cross-sectional study. Int J Women’s Health Reprod Sci. 2018;6(3):307–12. 10.15296/ijwhr.2018.51

[45] Chang SR, Yang CF, Chen KH. Relationships between body image, sexual dysfunction, and health-related quality of life among middle-aged women: a cross-sectional study. Maturitas. 2019 Aug;126:45–50. 10.1016/j.maturitas.2019.04.218 31239117

[46] Ellouze F, Bouzouita I, Chaari I, El Kefi H, Krir MW, Ben Cheikh C, et al. Relations entre sexualité, dépression et qualité de vie chez la femme Tunisienne enceinte. Sexologies. 2017;26(4):222–7. French. 10.1016/j.sexol.2017.06.002

[47] Oh EJ, Kim MJ. Factors affecting the sexual function of pregnant women. Korean J Women Health Nurs. 2019 Mar;25(1):73–85. 10.4069/kjwhn.2019.25.1.73 37679931

[48] Jalali T, Bostani Khalesi Z, Jafarzadeh-Kenarsari F. The association between sexual self-efficacy and the quality of life among menopausal women. J Menopausal Med. 2021 Aug;27(2):87–93. 10.6118/jmm.21006 34463072 PMC8408323

[49] Fasero M, Jurado-López AR, San Martín-Blanco C, Varillas-Delgado D, Coronado PJ. A higher quality of life by the Cervantes short-form scale is related to a better sexual desire in postmenopausal women. Gynecol Endocrinol. 2021 Nov;37(11):1014–9. 10.1080/09513590.2021.1929150 34018895

[50] Ghazanfarpour M, Khadivzadeh T, Babakhanian M. Investigating the relationship between sexual function and quality of life in menopausal women. J Family Reprod Health. 2016 Dec;10(4):191–7. 28546818 PMC5440818

[51] Peixoto C, Carrilho CG, Ribeiro TTSB, da Silva LM, Gonçalves EA, Fernandes L, et al. Relationship between sexual hormones, quality of life and postmenopausal sexual function. Trends Psychiatry Psychother. 2019 May 30;41(2):136–43. 10.1590/2237-6089-2018-0057 31166564

[52] Vedovo F, Di Blas L, Aretusi F, Falcone M, Perin C, Pavan N, et al. Physical, mental and sexual health among transgender women: a comparative study among operated transgender and cisgender women in a national tertiary referral network. J Sex Med. 2021 May;18(5):982–9. 10.1016/j.jsxm.2021.02.006 33771479

[53] Alidost F, Dolatian M, Shams J, Nasiri M, Sarkhoshpour E. The correlation of sexual dysfunction with prenatal stress and quality of life: a path analysis. Iran Red Crescent Med J. 2017;19(7): 10.5812/ircmj.55686

[54] Chatterjee SS, Bhattacharyya R, Chakraborty A, Lahiri A, Dasgupta A. Quality of life, sexual health, and associated factors among the sexually active adults in a metro city of India: an inquiry during the COVID-19 pandemic-related lockdown. Front Psychiatry. 2022 Mar 24;13:791001. 10.3389/fpsyt.2022.791001 35401271 PMC8987586

[55] Flynn TJ, Gow AJ. Examining associations between sexual behaviours and quality of life in older adults. Age Ageing. 2015 Sep;44(5):823–8. 10.1093/ageing/afv083 26178206

[56] Kim JS, Kang S. A study on body image, sexual quality of life, depression, and quality of life in middle-aged adults. Asian Nurs Res (Korean Soc Nurs Sci). 2015 Jun;9(2):96–103. 10.1016/j.anr.2014.12.001 26160236

[57] Najimi A, Veisani Y, Azami S, Azadi A. Investigating the sexual quality of life and its relationship with general health in older men in Iran. J Educ Health Promot. 2020 Jun 30;9(1):150. 10.4103/jehp.jehp_748_19 32766335 PMC7377141

[58] de Souza Júnior EV, Souza CS, Filho BFS, Siqueira LR, Silva CS, Sawada NO. Sexual function positively correlated with older adults’ sexuality and quality of life. Rev Bras Enferm. 2022 Aug 8;75(suppl 4):e20210939. 10.1590/0034-7167-2021-0939 35946725

[59] Eleuteri S, Giorgio M, Nesi G, March MR. The impact of depressive mood and sexual functioning in the quality of life of aging men. Int J Sex Health. 2019;31:A220.

[60] Lu Y, Fan S, Cui J, Yang Y, Song Y, Kang J, et al. The decline in sexual function, psychological disorders (anxiety and depression) and life satisfaction in older men: a cross-sectional study in a hospital-based population. Andrologia. 2020 Jun;52(5):e13559. 10.1111/and.13559 32162365

[61] Chao JK, Lin YC, Ma MC, Lai CJ, Ku YC, Kuo WH, et al. Relationship among sexual desire, sexual satisfaction, and quality of life in middle-aged and older adults. J Sex Marital Ther. 2011;37(5):386–403. 10.1080/0092623X.2011.607051 21961445

[62] Cabo JJ, Kaufman MR, Johnsen NV. Impact of sexual function domains on sexual satisfaction and quality of life: importance across the age spectrum. Andrology. 2024 Jan 16:andr.13594. 10.1111/andr.13594 38226963

[63] Schlichthorst M, Sanci LA, Hocking JS. Health and lifestyle factors associated with sexual difficulties in men - results from a study of Australian men aged 18 to 55 years. BMC Public Health. 2016 Oct 31;16(S3) Suppl 3:1043. 10.1186/s12889-016-3705-6 28185600 PMC5103242

[64] Holmberg D, Blair KL, Phillips M. Women’s sexual satisfaction as a predictor of well-being in same-sex versus mixed-sex relationships. J Sex Res. 2010 Jan;47(1):1–11. 10.1080/00224490902898710 19381998

[65] del Mar Sánchez-Fuentes M, Sierra JC. Sexual satisfaction in a heterosexual and homosexual Spanish sample: the role of socio-demographic characteristics, health indicators, and relational factors. Sex Relationship Ther. 2015;30(2):226–42. 10.1080/14681994.2014.978275

[66] Abedi P, Jorfi M, Afshari P, Fakhri A. How does health-promoting lifestyle relate to sexual function among women of reproductive age in Iran? Glob Health Promot. 2018 Sep;25(3):15–21. 10.1177/1757975917706831 28857683

[67] Smith JF, Breyer BN, Eisenberg ML, Sharlip ID, Shindel AW. Sexual function and depressive symptoms among male North American medical students. J Sex Med. 2010 Dec;7(12):3909–17. 10.1111/j.1743-6109.2010.02033.x 21059174 PMC3565609

[68] Shindel AW, Eisenberg ML, Breyer BN, Sharlip ID, Smith JF. Sexual function and depressive symptoms among female North American medical students. J Sex Med. 2011 Feb;8(2):391–9. 10.1111/j.1743-6109.2010.02085.x 21054793 PMC3565606

[69] Worsley R, Bell RJ, Gartoulla P, Davis SR. Prevalence and predictors of low sexual desire, sexually related personal distress, and hypoactive sexual desire dysfunction in a community-based sample of midlife women. J Sex Med. 2017 May;14(5):675–86. 10.1016/j.jsxm.2017.03.254 28499520

[70] Soler F, Granados R, Arcos-Romero AI, Calvillo C, Álvarez-Muelas A, Sánchez-Fuentes MDM, et al. Association between psychopathological dimensions and sexual functioning/sexual arousal in young adults. Int J Environ Res Public Health. 2021 Mar 30;18(7):3584. 10.3390/ijerph18073584 33808329 PMC8038005

[71] Effati-Daryani F, Jahanfar S, Mohammadi A, Zarei S, Mirghafourvand M. The relationship between sexual function and mental health in Iranian pregnant women during the COVID-19 pandemic. BMC Pregnancy Childbirth. 2021 Apr 26;21(1):327. 10.1186/s12884-021-03812-7 33902479 PMC8072090

[72] Ribeiro MC, Nakamura MU, Torloni MR, Scanavino MD, Mancini PE, Mattar R. Sexual function and quality of life of Brazilian pregnant women - preliminary results. J Sex Med. 2014;11:186.

[73] Tracy JK, Junginger J. Correlates of lesbian sexual functioning. J Womens Health (Larchmt). 2007 May;16(4):499–509. 10.1089/jwh.2006.0308 17521253

[74] Chang SR, Ho HN, Chen KH, Shyu MK, Huang LH, Lin WA. Depressive symptoms as a predictor of sexual function during pregnancy. J Sex Med. 2012 Oct;9(10):2582–9. 10.1111/j.1743-6109.2012.02874.x 22897117

[75] Nimbi FM, Tripodi F, Rossi R, Simonelli C. Expanding the analysis of psychosocial factors of sexual desire in men. J Sex Med. 2018 Feb;15(2):230–44. 10.1016/j.jsxm.2017.11.227 29292060

[76] Ryu SJ, Kwon M, Kim SA. Effects of depression, self-efficacy, and sexual function on quality of life in middle-aged Korean men. J Men’s Health. 2022;18(5):1. 10.31083/j.jomh1805108

[77] Pastuszak AW, Badhiwala N, Lipshultz LI, Khera M. Depression is correlated with the psychological and physical aspects of sexual dysfunction in men. Int J Impot Res. 2013 Sep;25(5):194–9. 10.1038/ijir.2013.4 23466661

[78] Przydacz M, Chlosta M, Chrobak AA, Rajwa P, Dudek P, Wiatr T, et al. Sexual activity in a large representative cohort of Polish men: frequency, number of partners, correlates, and quality of life. PLoS One. 2024 Jan 19;19(1):e0296449. 10.1371/journal.pone.0296449 38241234 PMC10798542

[79] Boyacıoğlu NE, Oflaz F, Karaahmet AY, Hodaeı BK, Afşin Y, Taşabat SE. Sexuality, quality of life and psychological well-being in older adults: a correlational study. Eur J Obstet Gynecol Reprod Biol X. 2023 Jan 12;17:100177. 10.1016/j.eurox.2023.100177 36718173 PMC9883179

[80] Jackson SE, Firth J, Veronese N, Stubbs B, Koyanagi A, Yang L, et al. Decline in sexuality and wellbeing in older adults: a population-based study. J Affect Disord. 2019 Feb 15;245:912–7. 10.1016/j.jad.2018.11.091 30699876

[81] Gil-Salmerón A, López-Sánchez GF, López-Bueno R, Pardhan S, Grabovac I, Smith L. Association between anxious and depressive symptomatology and sexual activity in Spain: a cross-sectional study during the COVID-19 quarantine. Int J Environ Res Public Health. 2021 Dec 23;19(1):147. 10.3390/ijerph19010147 35010405 PMC8751132

[82] Mollaioli D, Sansone A, Ciocca G, Limoncin E, Colonnello E, Di Lorenzo G, et al. Benefits of sexual activity on psychological, relational, and sexual health during the COVID-19 breakout. J Sex Med. 2021 Jan;18(1):35–49. 10.1016/j.jsxm.2020.10.008 33234430 PMC7584428

[83] Florkiewicz-Danel M, Zaręba K, Ciebiera M, Jakiel G. Quality of life and sexual satisfaction in the early period of motherhood-a cross-sectional preliminary study. J Clin Med. 2023 Dec 9;12(24):7597. 10.3390/jcm12247597 38137665 PMC10744264

[84] Muise A, Schimmack U, Impett EA. Sexual frequency predicts greater well-being, but more is not always better. Soc Psychol Personal Sci. 2016;7(4):295–302. 10.1177/1948550615616462

[85] Karakose S, Urs M, Marshall JE, Ledermann T. Depression, anxiety, stress, and sexual satisfaction in couples. J Sex Marital Ther. 2023;49(6):616–29. 10.1080/0092623X.2023.2166637 36688349

[86] Khorshidi M, Alimoradi Z, Bahrami N, Griffiths MD. Predictors of women’s sexual quality of life during the COVID-19 pandemic: an Iranian cross-sectional study. Sex Relatsh Ther. 2022;39(2):444–57. 10.1080/14681994.2022.2089645

[87] Gök Ç, Yücel U, Okuyan YÇ, Akmeşe ZB. Impact of perceived social support and depression in married Turkish women on the sexual quality of life: an online survey. Niger J Clin Pract. 2023 Nov 1;26(11):1667–76. 10.4103/njcp.njcp_293_23 38044772

[88] Oveisi N, Khan Z, Brotto LA. Relationship of sexual quality of life and mental well-being in undergraduate women in a Canadian university. Can J Hum Sex. 2022;31(3):422–31. 10.3138/cjhs.2022-0012

[89] Wåhlin-Jacobsen S, Kristensen E, Pedersen AT, Laessøe NC, Cohen AS, Hougaard DM, et al. Androgens and psychosocial factors related to sexual dysfunctions in premenopausal women∗: ∗2016 ISSM female sexual dysfunction prize. J Sex Med. 2017 Mar;14(3):366–79. 10.1016/j.jsxm.2016.12.237 28117267

[90] Tavares IM, Rosen NO, Heiman JR, Nobre PJ. Biopsychosocial predictors of couples’ trajectories of sexual function and sexual distress across the transition to parenthood. Arch Sex Behav. 2023 May;52(4):1493–511. 10.1007/s10508-022-02480-8 36459350

[91] Bahrami N, Hosseini M, Griffiths MD, Alimoradi Z. Sexual-related determinants of life satisfaction among married women: a cross-sectional study. BMC Womens Health. 2023 Apr 28;23(1):204. 10.1186/s12905-023-02365-5 37118721 PMC10148412

[92] Debrot A, Meuwly N, Muise A, Impett EA, Schoebi D. More than just sex: affection mediates the association between sexual activity and well-being. Pers Soc Psychol Bull. 2017 Mar;43(3):287–99. 10.1177/0146167216684124 28903688

[93] Davison SL, Bell RJ, LaChina M, Holden SL, Davis SR. The relationship between self-reported sexual satisfaction and general well-being in women. J Sex Med. 2009 Oct;6(10):2690–7. 10.1111/j.1743-6109.2009.01406.x 19817981

[94] Dogan T, Tugut N, Golbasi Z. The relationship between sexual quality of life, happiness, and satisfaction with life in married Turkish women. Sex Disabil. 2013;31(3):239–47. 10.1007/s11195-013-9302-z

[95] Pollard AE, Rogge RD. Love in the Time of COVID-19: A Multi-Wave Study Examining the Salience of Sexual and Relationship Health During the COVID-19 Pandemic. Arch Sex Behav. 2022 Jan;51(1):247–71. 10.1007/s10508-021-02208-0 35083594 PMC8791703

[96] Rosen R, Brown C, Heiman J, Leiblum S, Meston C, Shabsigh R, et al. The female sexual function index (FSFI): a multidimensional self-report instrument for the assessment of female sexual function. J Sex Marital Ther. 2000 Apr-Jun;26(2):191–208. 10.1080/009262300278597 10782451

[97] Rosen RC, Cappelleri JC, Smith MD, Lipsky J, Peña BM. Development and evaluation of an abridged, 5-item version of the international index of erectile function (IIEF-5) as a diagnostic tool for erectile dysfunction. Int J Impot Res. 1999 Dec;11(6):319–26. 10.1038/sj.ijir.3900472 10637462

[98] Rosen RC, Riley A, Wagner G, Osterloh IH, Kirkpatrick J, Mishra A. The international index of erectile function (IIEF): a multidimensional scale for assessment of erectile dysfunction. Urology. 1997 Jun;49(6):822–30. 10.1016/S0090-4295(97)00238-0 9187685

[99] DeRogatis LR, Rosen R, Leiblum S, Burnett A, Heiman J. the female sexual distress scale (FSDS): initial validation of a standardized scale for assessment of sexually related personal distress in women. J Sex Marital Ther. 2002 Jul-Sep;28(4):317–30. 10.1080/00926230290001448 12082670

[100] DeRogatis L, Clayton A, Lewis-D’Agostino D, Wunderlich G, Fu Y. Validation of the female sexual distress scale-revised for assessing distress in women with hypoactive sexual desire disorder. J Sex Med. 2008 Feb;5(2):357–64. 10.1111/j.1743-6109.2007.00672.x 18042215

[101] World Health Organization. Introduction to the WHO common framework on measuring and reporting on the health of populations. Geneva: United Nations Economic Commission for Europe; 2000. Available from: https://unece.org/fileadmin/DAM/stats/documents/ece/ces/2000/10/health/wp.7.e.pdf [cited 2024 Jul 15].

[102] The World Health Organization quality of life assessment (WHOQOL): position paper from the World Health Organization. Soc Sci Med. 1995 Nov;41(10):1403–9. 10.1016/0277-9536(95)00112-K 8560308

[103] Power M, Quinn K, Schmidt S, WHOQOL-OLD Group. Development of the WHOQOL-old module. Qual Life Res. 2005 Dec;14(10):2197–214. 10.1007/s11136-005-7380-9 16328900

[104] Ware JE Jr, Sherbourne CD. The MOS 36-item short-form health survey (SF-36). I. Conceptual framework and item selection. Med Care. 1992 Jun;30(6):473–83. 10.1097/00005650-199206000-00002 1593914

[105] Ware J Jr, Kosinski M, Keller SDA. A 12-item short-form health survey: construction of scales and preliminary tests of reliability and validity. Med Care. 1996 Mar;34(3):220–33. 10.1097/00005650-199603000-00003 8628042

[106] Barger D. Sexual function and quality of life: assessing existing tools and considerations for new technologies. In: Wac K, Wulfovich S, editors. Quantifying quality of life. Health informatics. Cham: Springer International Publishing; 2022. pp. 395–427. 10.1007/978-3-030-94212-0_16

[107] Lewis R, Bosó Pérez R, Maxwell KJ, Reid D, Macdowall W, Bonell C, et al. Conceptualizing sexual wellbeing: a qualitative investigation to inform development of a measure (Natsal-SW). J Sex Res. 2024 Mar 22; (Mar):1–19. 10.1080/00224499.2024.2326933 38517458 PMC7619709

[108] Sánchez-Fuentes MM, Santos-Iglesias P, Sierra JC. A systematic review of sexual satisfaction. Int J Clin Health Psychol. 2014;14(1):67–75. 10.1016/S1697-2600(14)70038-9

[109] Gore-Gorszewska G. “What do you mean by sex?” A qualitative analysis of traditional versus evolved meanings of sexual activity among older women and men. J Sex Res. 2021 Oct;58(8):1035–49. 10.1080/00224499.2020.1798333 32779942

[110] Narasimhan M, Say L, Allotey P. Three decades of progress and setbacks since the first international conference on population and development. Bull World Health Organ. 2024;102(4):226–226a. 10.2471/BLT.24.291654 38562203 PMC10976866

[111] Ghebreyesus TA, Allotey P, Narasimhan M. Advancing the “sexual” in sexual and reproductive health and rights: a global health, gender equality and human rights imperative. Bull World Health Organ. 2024 Jan 1;102(1):77–8. 10.2471/BLT.23.291227 38164333 PMC10753275

[112] Ford JV, Corona-Vargas E, Cruz M, Fortenberry JD, Kismodi E, Philpott A, et al. The world association for sexual health’s declaration on sexual pleasure: a technical guide. Int J Sex Health. 2022 Jan 25;33(4):612–42. 10.1080/19317611.2021.2023718 38595778 PMC10903694

